# Testis Transcriptome Modulation in Klinefelter Patients with Hypospermatogenesis

**DOI:** 10.1038/srep45729

**Published:** 2017-03-31

**Authors:** Marco D’Aurora, Alberto Ferlin, Andrea Garolla, Sara Franchi, Laura D’Onofrio, Oriana Trubiani, Giandomenico Palka, Carlo Foresta, Liborio Stuppia, Valentina Gatta

**Affiliations:** 1 Department of Psychological, Health and Territorial Sciences, School of Medicine and Health Sciences, “G.d’Annunzio” University, Via Dei Vestini 31, 66100, Chieti, Italy; 2 Functional Genetics Unit, Center of Excellence on Aging (Ce.S.I.), Via Dei Vestini 31, 66100, Chieti, Italy; 3 Department of Medicine, Unit of Andrology and Reproductive Medicine, University of Padova, Via Giustiniani 2, 35128, Padova, Italy; 4 Department of Oral Health and Biotechnological Sciences, School of Medicine and Health Sciences, “G.d’Annunzio” University, Via DeiVestini 31, 66100, Chieti, Italy

## Abstract

The main genetic cause of male infertility is represented by the Klinefelter Syndrome (KS), a condition accounting for 3% of all cases of infertility and up to15% of cases of azoospermia. KS is generally characterized by azoospermia; approximately 10% of cases have severe oligozoospermia. Among these, the 30–40% of patients show hypospermatogenesis. The mechanisms leading to adult testis dysfunctions are not completely understood. A microarray transcriptome analysis was performed on testis biopsies obtained from three KS patients with hypospermatogenesis and three control subjects. KS testis showed a differential up- and down-regulation of 303 and 747 transcripts, respectively, as compared to controls. The majority of down-regulated transcripts were involved in spermiogenesis failure and testis morphological defects, whereas up-regulated genes were responsible for testis apoptotic processes. Functional analysis of the transcriptionally altered genes indicated a deregulation in cell death, germ cell function and morphology as well as blood-testis-barrier maintenance and Leydig cells activity. These data support a complex scenario in which spermatogenic impairment is the result of functional and morphological alterations in both germinal and somatic components of KS testis. These findings could represent the basis for evaluating new markers of KS spermatogenesis and potential targets of therapeutic intervention to preserve residual spermatogenesis.

The Klinefelter Syndrome (KS) is the most common aberration of sex chromosomes, with a prevalence of 1:600–1:1000 in newborn males, although it is generally diagnosed in the adulthood^1^. KS represents the main genetic cause of male infertility being present in 3% of infertile men and 15% of azoospermic men^2^. The majority of KS subjects (80–90%) show a non-mosaic 47,XXY karyotype, whereas the remaining 10% of cases carry mosaicisms (e.g. 47,XXY/46,XY), additional sex chromosomes (e.g. 48,XXXY, 48,XXYY, 49,XXXXY) or X chromosome structural abnormalities (e.g. 47,X,iXq,Y)^3^. KS patients are traditionally described as azoospermic in approximately 90% of cases, evidencing primary testicular failure, small firm testes and hypergonadotropic hypogonadism^4^, however, the phenotype is often highly variable. A very low percentage of KS patients (10%) is described with severe oligozoospermia. About 30–40% of KS patients has hypospermatogenesis in the testis. In light of this, it is suggested that focal spermatogenesis has thecapability of completing the spermatogenic process leading to the formation of mature spermatozoa^5^,^6^,^7^.

KS-related azoospermia or oligozoospermia are the consequences of the progressive degeneration of germinal epithelium that starts early in embryo life. The differentiation process begins only when germ cells show a normal karyotype^8^. During mid-puberty, testicular functions move towards an extensive impairment^9^ that is associated with fibrosis and hyalinization of the seminiferous tubules and the hyperplasia of the interstitium^8^.

The molecular mechanisms underlying the dysfunction in KS testis have been poorly investigated and the causes of global testicular degeneration are still unclear. The presence of an additional X-chromosome seems to significantly impair spermatogenesis at an early stage^10^,^11^. In meiotic and post-meiotic germ cells as well as in somatic components of the testis, the extra X chromosome is inactive and the altered expression of genes located in the autosomes might be the cause of the global testis dysfunction^10^,^11^.

Nowadays, the fertility therapy available for azoospermic KS men is based on TESE (Testicular Sperm Extraction after Biopsy) and assisted reproduction^12^. The molecular networks involved in KS spermatogenic and testicular failure could represent the basis for the identification of new therapy targets and the set-up of early treatments preserving residual fertility.

Our earlier transcriptomic study on adult azoospermic KS patients, showing complete absence of germ cells (Sertoli cell only syndrome, SCO) offered clue to probable mechanisms leading to testis dysfunction^13^. Similarly, this pilot innovative study analyze the entire transcriptomic modifications occurring in adult KS testis having hypospermatogenesis. The comparison of the results from the two studies highlights the similarities and the differences between azoospermic and hypospermatogenic KS subjects.

## Results

**Figure 1:**
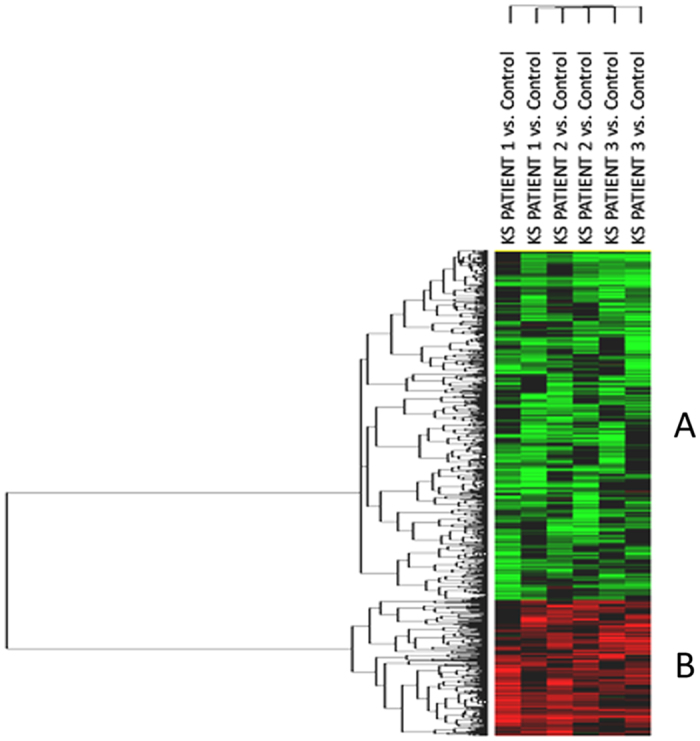
Unsupervised hierarchical clustering of significant genes. The heatmap shows the differential expression of the 1050 significantly expressed genes in mosaic KS testis vs. control testis, grouped in two different clusters composed respectively by 747 down-regulated genes (Cluster A) and 303 up-regulated transcripts (Cluster B). The top tree represents the 6 experiments (3 patients run as a duplicate dye-swap). Each patient RNA was analysed against the control RNA (pool of RNA from three control subjects). In green the genes showing negative expression, in red the genes showing positive expression. Black and grey represent respectively missing and not significant changes in expression.

Transcriptome analysis revealed the differential expression of 1050 genes as compared to control testis. The heat-map of the significant transcripts was formed by two differentially expressed gene clusters (Clusters A and B) composed of 747 down- and 303 up-regulated genes in hypospermatogenesis KS testis vs. control testis (Fig. 1, Supplementary Table S1 and S2).

**Figure 2:**
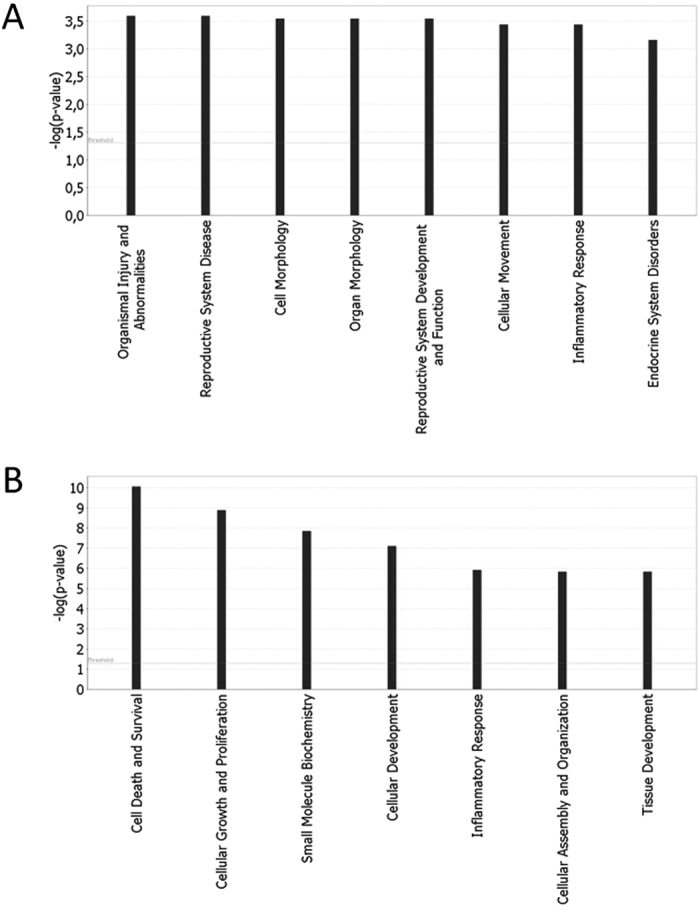
IPA-inferred functional analysis of cluster A and B gene datasets. IPA functional analysis of cluster A and B datasets revealed the main functions modulated by differentially expressed transcripts. (**A**) Bar chart shows the key biological functions associated to cluster A dataset. (**B**) Bar chart shows the key biological functions associated to cluster B dataset. The −log (p-value), is calculated by IPA algorithm based on the number of genes involved in the function and their reported role.

Functional Ingenuity Pathway Analysis (IPA) of the two gene clusters revealed that the 747 down-regulated genes were mainly involved in the following biological functions: organismal injury and abnormalities, reproductive system disease, cell and organ morphology, reproductive system development and function, cellular movement, inflammatory response and endocrine system disorders (Fig. 2A). The 303 over-expressed genes are known for their role in the following biological functions: cell death and survival, cellular growth and proliferation, small molecule biochemistry, cellular development, inflammatory response, cellular assembly and organization and tissue development (Fig. 2B).

**Figure 3:**
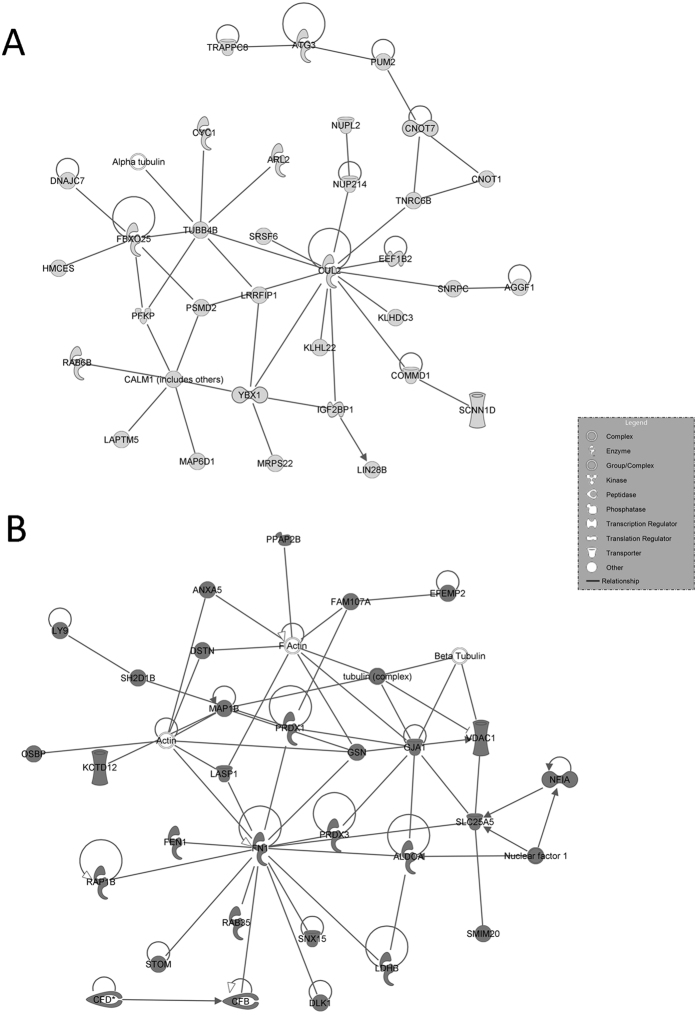
IPA-inferred top-scored networks associated to cluster A and B gene datasets. IPA-inferred network analysis was generated for clusters A and B transcripts linking genes functionality to disclose mechanistic networks based on their connectivity and enrichment statistics. (**A**) Top gene network generated by IPA for Cluster A. The network is centred around the key node genes CUL2 and CALM1, involved in pathways that regulate the communications between germ and Sertoli cells. (**B**) Top gene network generated by IPA for Cluster B. The network shows the involvement of FGFs and NFKb pathways in increased testicular apoptosis. In light grey the genes down-regulated listed in cluster A, in dark grey the genes up-regulated listed in cluster B. White genes are transcripts not modulated in our series.

**Figure 4:**
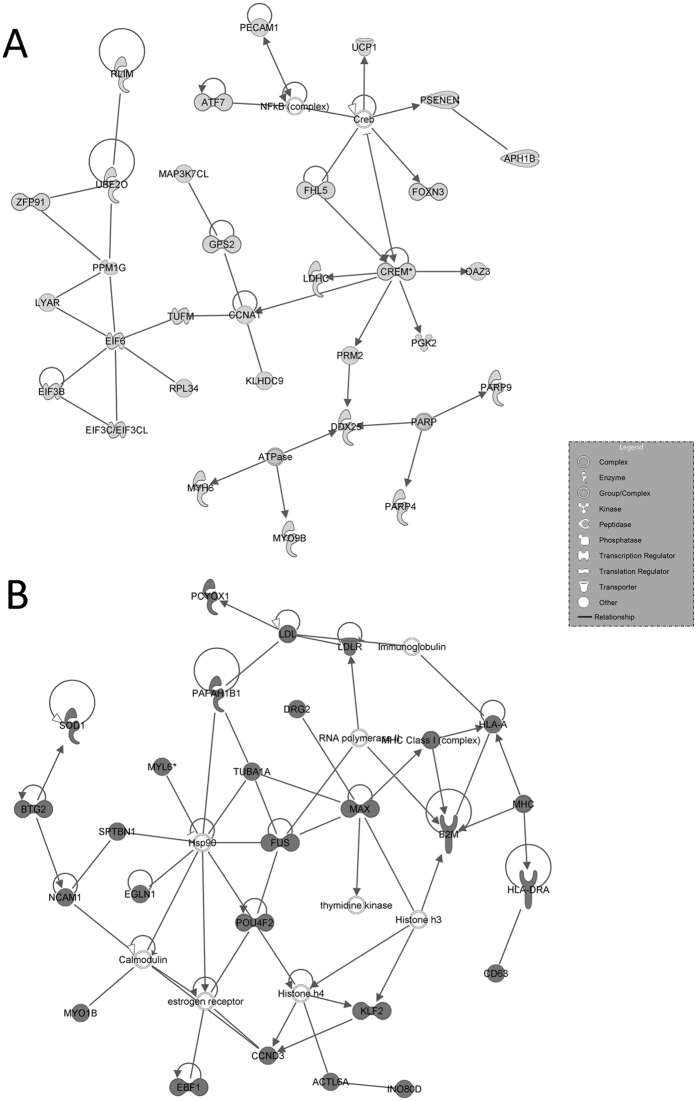
IPA-inferred second-scored networks associated to cluster A and B gene datasets. (**A**) Second-scored gene network generated by IPA for Cluster A. The network is centred around the key node CREM, main testicular transcription factor essential to spermatozoa production. (**B**) Second-scored gene network generated by IPA for Cluster B. The network shows the presence of transcripts causing increased inflammation and necrosis processes. In light grey the genes down-regulated listed in cluster A, in dark grey the genes up-regulated listed in cluster B. White genes are transcripts not modulated in our series.

IPA-inferred network analysis on the data set indicated 25 networks for cluster A with a score ranging from 56 down to 16, and16 networks for cluster B with a score ranging from 56 down to 13. The two top-scored networks associated to each cluster are presented in Figs 3 and 4.

The analysis of the deregulated datasets indicates a number of mechanisms involved in the hypospermatogenesis state of adult KS, such as: (i) inability of spermatogonia to differentiate into mature spermatozoa; (ii) morphological and structural defects of testis; (iii) physiological alterations in the testicular environment; (iv) extensive apoptosis of germinal and somatic components.

Four genes in cluster A (CREM, AKAP4, LDHC and SPA17) and 4 genes in cluster B (BCL2, GSTA2, JUN and SOD1) were validated by quantitative Real-Time PCR (qRT-PCR). In each sample, the selected genes presented significant fold differences in their relative expression when compared with the expression in the control testis samples (p < 0.05, Student’s T-test). qRT-PCR analysis confirmed microarray data showing no significant fold change differences (p > 0.05, Student’s T-test) (Supplementary Figure S1).

Microarray results were confirmed by Western Blots for JUN and BCL2 (up-regulated) and SPA17 (down-regulated) proteins, obtained from the same patients and controls enrolled for the study. (Supplementary Figure S2).

## Discussion

The presence of an extra X-chromosome can explain the crucial role played by the expression of a surplus of X-linked genes in KS patients during the early stage of spermatogenesis^14^ and gives rise to a series of events defining the adult testis failure, but their role is marginal in adult testis functions. We could narrow down to the over-regulation of 3 genes (SLC25A5 on Par1 region, PRPS1, TCS22D3), and the down-regulation of the AKAP4 gene, mapped on the X chromosome. Several autosomal transcripts involved across the whole spermatozoa production events appear to be involved in the testis dysregulation leading to hypospermatogenesis in adult KS subjects.

Our microarray analysis showed the down-regulation of CREM. CREM is the main testicular transcription factor essential to spermatozoa production^15^ and development of seminiferous tubules. CREM is the key node of IPA-inferred second network (Fig. 4A), which negatively regulates the expression of other essential spermatogenesis factors such as FHL5 and PRM1 and PRM2. FHL5 is a component of the CREM pathway regulating germ cells meiosis^16^, while PRM1 and PRM2 encode the proteins involved in the replacement of histones with protamines during spermiogenesis. It has been recently reported that the altered amount of PRM1 and PRM2 transcripts influences the whole spermiogenesis process and the sperm morphology, thereby affecting the functions of mature sperm^17^,^18^. The expression levels of PRM1 and PRM2 genes are reported to be indicators of male infertility^18^.

The IPA analysis showed the down-regulation of eight genes specifically influencing the male meiosis. Among them, HORMAD2 is known to be essential during meiotic prophase^19^, while CCNA1 is a meiosis-specific cyclin required for meiosis I in testicular germ epithelium^20^. The relative abundance of CCNA1 transcript was reported to be lower in patients with severe defects of sperm production^20^. Network analysis identified another key gene, EZH2. This gene has a role in the self-renewal of primordial germ cells^21^ and its activity is directly correlated with the transcription factors YBX1, YBX2 and MLH1 that regulate germ cell differentiation and meiosis^22^. EZH2 regulates the expression of KNAP2, that occurs in testicular germ cells late in spermatogenesis^23^. In addition, the expression of classical sperm markers (SPAG5, SPAG9, SPA17, SPATA8, SPATA17, SPATA22, SPATA24 and SPATS2L) was found down-regulated. This associates with meiosis arrest, infertility and germ cells apoptosis^24^,^25^,^26^,^27^. These data indicate that the defects in meiosis and germ cells differentiation could be responsible for the reduced number of mature spermatozoa in KS patients.

The IPA analysis of the down-regulated genes dataset revealed that many transcripts are related to hypospermatogenic status, such as CNOT7, FKBP4 or RLN1. These genes were reported to be down-regulated in infertility conditions and spermatogenesis alterations in mice^28^,^29^,^30^, and could be used as markers of hypospermatogenic testis.

The down-regulated genes could mainly be expressed in germ cells while the up-regulated genes are likely expressed in somatic components, suggesting a differential implication of these genes in KS pathogenic context (Supplementary Table S1 and S2). Several down-regulated transcripts are linked to germ cells morphology and movement. The down-modulation of AKAP4, CNOT7, DDX25, FHL5, NR1H3, FHL5, PARVB, PRND, SEPT12, SPESP1, SYCP3, and TAF4B transcripts could be associated to the abnormal morphology of germ cells. In particular, TAF4B is a post-meiotic transcription factor that regulates spermiogenesis, and its disruption was associated to structurally abnormal sperm, reduced sperm count, and reduced motility^31^. AKAP4 (located on the X chromosome) encodes a structural protein of mature spermatozoa tails. Its lower expression could be linked to reduced sperm motility^32^.

Other down-regulated transcripts such as FKBP4, GAPDHS, LDHC, PGK2, SLC2A8, and TRPV6 are reported by IPA analysis to play a central role in male germ cells movement, an essential feature for the correct sperm maturation and the release into testis luminal edge. This movement is associated to morphological changes and is strictly controlled by Sertoli cells (SCs)^33^. Once released, the mature spermatozoa acquire tail functionality, a process that requires energy production by mitochondria. FKBP4, GAPDHS, LDHC, PGK2, SLC2A8, and TRPV6 act specifically during germ maturation across SCs structure and are involved in the tail functionality^34^,^35^,^36^. Several transcripts in the down-regulated genes dataset encode mitochondrial-related proteins (MRPs, MTCH2, mitochondrial solute carriers, TCAIM, TUFM, UCP1, and CMC2). These data suggest the inability of KS germ cell to acquire movement for a complete maturation process.

Failure of germ cell maturation is in consonance with the altered expression of many genes involved in SCs function. It is well known that SCs structure and metabolic activity are essential to germ cells differentiation. Our results show the deregulation of many genes encoding membrane proteins as well as anchoring and cytoskeleton proteins that are important for SCs functionality, promoting the inability of SCs to sustain germ cells development and to maintain Blood-Testis-Barrier (BTB) integrity. A down-regulation of several actins, AJAP1, COL13A1, MASTL, protocadherins, TPGS2, TPPP2, TTL, TTLL6, TUBB4B, and TUBGCP5, was identified whereas CAV1, CDH13, FN1, SMIM4, TMC8, TMED10P1, TMEM79, TBCA and TUBA1A were found to be up-regulated. These modifications could reflect defects in SCs shape and function, in microtubule dynamics as well as in the mitotic and meiotic division of germ cells^37^. The top network associated to Cluster A (Fig. 3A), centered around CUL2 and CALM1 nodes, clearly draws alterations in the inter-communications between germ cells and SCs. In addition, results show the differential expression of many transcripts involved in SCs molecular transport and metabolic activity that are key features of BTB maintenance and germinal development^38^, and explains the “nurse-role” of SCs. SCs metabolites and small inorganic molecules are transported to growing germ cells through specialized channels^39^. Two clear examples are the transport of zinc and glucose/lactate^40^. Data highlights the down regulation of many solute carriers such as the zinc transporter SLC39A3 or glucose transporters SLC2A5 and SLC2A8. Noteworthy, our study identified the down-regulation of LDHAL6B and LDHC, two genes encoding for lactate metabolism enzymes. Germ cells use lactate produced by SCs as their main energy source^41^, and our findings confirm that SCs are unable to properly sustain the germ cells.

The analysis of the dataset on up-regulated genes provides evidence for the modification of transcripts expressed in somatic components that could be responsible for their apoptosis and hyper-activation of Leydig cells (LCs).

KS patients show classically a hypergonadotropic hypogonadism due to primary testicular insufficiency. FSH is the main hormone regulating spermatogenesis acting through SCs, whereas LH stimulates testosterone production by the LCs. The altered expression of genes regulated by FSH and LH pathway was found. The up-regulation of INHBA and ANXA5 affects the FSH level and the hypotalamo-pitutitary axis respectively, regulating the testosterone production^42^,^43^. Thirty-one down-modulated transcripts participate in the regulation of “Endocrine system disorders” (Fig. 2A). In Fig. 3A, CUL2 is linked to IGF2BP, a regulator of translation. It is known that the members of the insulin growth factors pathway are regulated by FSH action^44^. We believe that the expression of the transcripts modulated in SCs and LCs could be directly or indirectly linked to FSH and LH levels. The up-regulation of Sertoli and Leydig cell genes, probably due to hyperactivation of these cells, was found in KS patients with Sertoli cell-only syndrome (SCO)^13^ and hyperplasia of Leydig cells is a common finding in KS subjects. However, whether this dysregulation is essentially due to higher FSH and LH or to a primary testicular damage, being responsible for increased levels of gonadotropins, remains an open question.

Of the 303 up-regulated transcripts, one third were linked to apoptotic processes (Supplementary Table S3). Among these genes, IPA revealed the up-regulation of both pro and anti-apoptotic genes. Given the extensive death of germinal cells, somatic components apoptosis disturbance could represent the main mechanism leading to KS testis dysfunction^1^. Many genes are expressed in both somatic and germinal cells, thus it is difficult to distinguish the specific effects of their up-regulation. Two of the most important genes in the up-regulated dataset are JUN, which regulates LCs apoptosis, depending on the androgens production^45^ and BCL2, whose up-regulation could be a reaction to the mechanisms which induce apoptosis^46^. Apoptosis seems also to be linked to the FGFs and NFKb pathways, as depicted by IPA-inferred top network in Fig. 3B. These networks are reported to regulate male fertility ensuring somatic/germinal cells functionality and development^47^. The up-regulation of these apoptotic pathways may be due to an imbalance between apoptotic and survival signals in both somatic and germinal components. Our study’s results suggest that apoptosis in adult testis could be a consequence of germ cell loss. Apoptosis of germ cells could as well result from failure of surrounding cells in providing optimal conditions for their growth. It is difficult to assess whether apoptosis inhibition should revert the situation, but it sounds possible that apoptosis block could ameliorate in part the pathological condition in KS testis. To our knowledge, no earlier reports have directly addressed the possible therapeutic inhibition of apoptosis to revert the situation. However, Aksglæde and colleagues reported that in the human testis, testosterone is able to effectively inhibit *in vitro*-induced apoptosis of spermatocytes and spermatids as well as estrogens effectively inhibit male germ-cell apoptosis in the cultured human seminiferous tubules^1^.

KS testis seem also to be affected by inflammation and necrosis processes, leading to cellular death and infertility^48^. IPA analysis of gene network 2 (Fig. 4B) illustrates the above mentioned features, reporting known genes of the inflammatory pathway (CDC42, HLA-DRA complex, IL1R1, SOD1, and SOCS3). It is worth noting that genes involved in redox homeostasis such as GSTA2 and GPX1 are up-regulated, and likely a sign of inflammation and LCs hyper activation^49^,^50^.

A study from Ma *et al*.^51^ reported the gene expression profile of induced pluripotent stem cells (iPSCs) obtained from a KS patient. Our results are in line with those reported in this study showing the modulation of genes which participate in the inflammatory and apoptotic pathways. On the contrary, KS-iPSCs showed the up-modulation of a high number of X-linked transcripts that were undetected in our study^51^, confirming that X-linked genes could have a role during the early stage of spermatogenesis that leads to the testicular dysfunctions in adult KS patients.

The results of our study were compared with those previously reported for KS azoospermic patients (SCO phenotype)^13^ to identify the molecular networks involved in KS spermiogenic and testicular failure. KS azoospermic testis showed the differential up- and down-regulation of 656 and 247 transcripts respectively, as compared to the same controls. As previously reported, the majority of modulated transcripts were expressed by Sertoli and Leydig cells. Data sets analysis indicate expression changes of genes playing a central role in cell death, inflammatory response, lipid metabolism, steroidogenesis, blood-testis-barrier formation and maintenance, as well as spermatogenesis failure.

**Figure 5:**
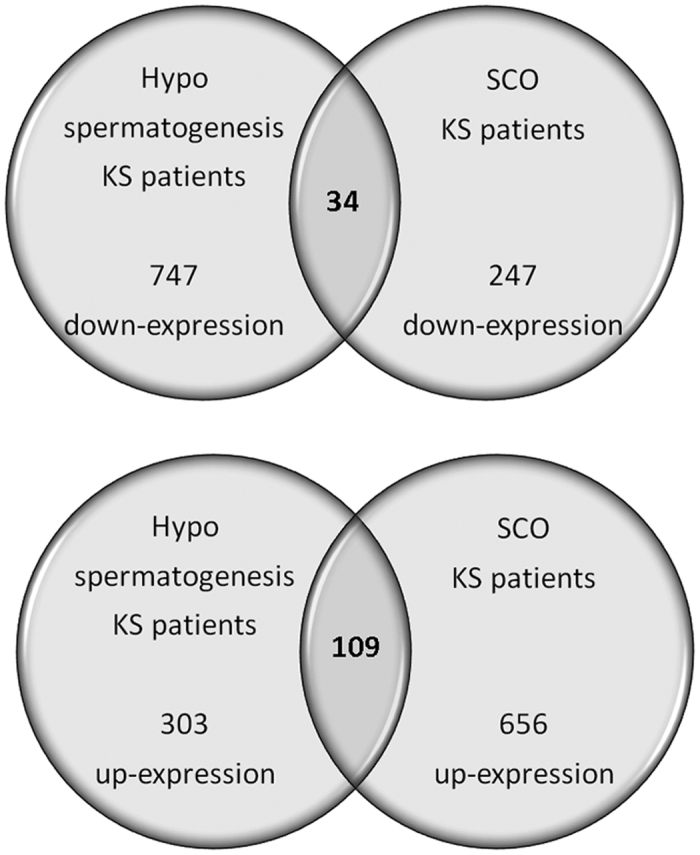
Common transcripts shared by KS patients affected by SCO and hypospermatogenesis. The figure reports the transcripts that show similar gene expression in Klinefelter patients affected by SCO or hypospermatogenesis when compared to control testis. The up-regulated transcripts are mainly involved in apoptosis, while the down-regulated transcripts are strictly linked to the absence (SCO) or heavy reduced number (hypospermatogenesis) of germ cells.

The comparison between patients with hypospermatogenesis and SCO revealed that 109 up-regulated and 34 down-regulated transcripts were shared by the two different conditions (Supplementary Table S4 and S5). The functional analysis of the 109 up-regulated genes reveals that they are involved in intercellular interaction, regulation of blood-testis-barrier, as well as apoptosis. This confirms the general KS testis dysfunction in both the hypospermatogenesis and SCO conditions. On the contrary, only 34 down-regulated genes are shared between the two conditions. The low number (n = 34) of shared down-regulated transcripts depends on the low number of down-regulated transcripts highlighted for patients with SCO (Fig. 5)^13^. In fact, KS patients affected by hypospermatogenesis, show a higher number of germ cells when compared to SCO patients, allowing the detection of a higher number of genes expressed in the germinal epithelium. These transcripts are down-regulated in KS patients with hypospermatogenesis when compared to normal controls (see Fig. 1, Cluster A).

## Conclusions

In conclusion, our study indicates a complex scenario in which spermatogenic impairment seems the result of functional and morphological alterations in both germinal and somatic components of KS testis with hypospermatogenesis. Published results show a specific expression pattern related to the clinical phenotype^52^. In addition, our results depict a picture which is similar to that reported by Zhuang *et al*., in non-obstructive azoospermic (NOA) patients^53^. On the other hand, the correct gene expression in germ cells drives the residual spermatogenesis following the modulation of several transcripts involved in germ cells morphology and movement as well as in the somatic component functions. In addition, data confirm the importance of the testis microenvironment integrity to sustain the germ cell growth that appears remarkably in KS.

## Future Perspectives

Further target studies are needed to validate these results in a larger population, to identify useful infertility markers and to remove interpersonal patients differences. However, these data could represent the basis to uncover molecular pathways that cause KS testis failure. Few studies reported expression modifications in blood samples from KS patients^54^, concluding that peripheral blood analysis could be representative of disease progression and likely could be used as a personalized non-invasive tool for differential diagnosis in patients showing variable phenotypes. Our results support this hypothesis, revealing that some genes are involved in pathways that are not peculiar to testis (i.e. Diabetes Mellitus Signaling, Neurological Disorders or Cardiovascular Disease). This may suggest the possibility that, for some genes, a similar expression modifications could be found in other organs or tissues. Blood expression analysis could help to differentiate the changes that are testis-specific, to highlight definite biomarkers for intervention. It would also be challenging to look at the modifications in non-coding RNAs (ncRNAs) expression to have a global view of the mechanisms altered in KS testis and likely the causes of altered gene expression. In addition, it would be noteworthy to study ncRNAs originating from X chromosome that could escape X inactivation.

## Methods

### Patients

The study was approved by the local Ethics Committee of the University Hospital of Padova and was in accordance with the Helsinki II Declaration. All participants were asked for and provided their informed consent. Bilateral testicular biopsies were obtained from 3 non-mosaic 47,XXY KS patients (mean age 29.3 ± 3.2) affected by azoospermia (absence of sperm in the ejaculate) and hypospermatogenesis (all germ cell stages present including spermatozoa, but with distinct decline in the number of germ cells) in the testes and from 3 controls subjects (mean age 29.1 ± 4.4) with obstructive azoospermia, normal spermatogenesis and normal 46,XY karyotype. Details of method and processing of testicular biopsies are described in our previous study^55^. All KS patients had a non-mosaic 47,XXY karyotype as evidenced by cytogenetic investigation on at least 50 metaphases, had not received testosterone substitution prior to biopsy and had negative history for other possible causes of testicular damage. Main clinical characteristics of Klinefelter cases and controls are shown in Table 1.

### Microarray analysis

Total RNA was extracted from homogenized testicular biopsies using the RNeasy^®^ Microarray Tissue Mini Kit (Qiagen, Hilden, Germany), following manufacturer instructions. Total purified RNA was linearly amplified using the AminoAllylMessageAmp™ II aRNA Amplification Kit (Ambion, Austin, TX, USA), fluorescently labelled with Cy3 or Cy5 cyanins, and then hybridized on high-density arrays HOA_005 Human Whole Genome OneArray™ Microarray V5 (Phalanx Biotech, Belmont, CA, USA), containing 29,187 human probes. Each array experiment was repeated as a dye-swap, for a total of 6 experiments.

Detailed processing and normalization of microarray raw data were described in our previous study^13^.

Raw data of the performed experiments have been recorded in the GEO public database (accession number: GSE83989).

### Statistical analysis

Raw data underwent a statistical analysis by R. Only those genes showing an absolute fold change (FC) <0.7 or >1.4, a present call in at least the 50% of experiments and a p-Value < 0.05 (ANOVA test) were considered significantly expressed. False Discovery Rate (FDR) was used to adjust p-values and to correct for the multiple testing issues^56^; FDR was calculated by R and considered significant when <10%. The resulting gene lists underwent a clustering analysis (Cluster 3.0 and TreeView, Stanford University Labs) to unravel Genes Differentially Expressed (DEGs) in mosaic KS patients testis vs. control testis. The identified up- and down-regulated transcripts were analysed by Ingenuity Pathways Analysis (IPA) software (Ingenuity Systems, Redwood City, CA, USA) to disclose the biological functions and the functional networks they are involved in. Biological functions are the measure of the likelihood that the association between the genes dataset and a related function is due to random association. IPA-generated p-Values < 0.05 indicate a statistically significant, non-random association. A Fisher’s exact test was carried out to generate network scores, based on the number of eligible molecules and their size and the total number that could be included in a network.

### qRT-PCR microarray data validation

The deregulated expression of 8 microarray genes (CREM, AKAP4, LDHC, SPA17, BCL2, GSTA, JUN, SOD1) was evaluated by quantitative Real-Time PCR (qRT-PCR). Specific primer and probe sets employed were purchased from ThermoFisher Scientific (Waltham, MA, USA): CREM Hs01590456, AKAP4 Hs00275849, LDHC Hs00255650, SPA17 Hs01011126, BCL2 Hs00608023, GSTA Hs00747232, JUN Hs01103582, SOD1 Hs00533490.

Three μg of total purified RNA were reverse transcribed using the High Capacity RNA-to-cDNA Kit (ThermoFisher Scientific, Waltham, MA, USA). qRT-PCR was performed in a total volume of 20 μl containing KAPA Probe Fast Abi Prism qPCR Kit (KAPA Biosystems), 25 ng of cDNA and 1 μl of primer-probe mixture (20X) on an Abi 7900HT Sequencing Detection System. The selected genes relative expression was corrected against GAPDH (Hs02758991) and HPRT1 (Hs02800695) were used as endogenous controls (ThermoFisher Scientific, Waltham, MA, USA). Real time amplification conditions were: 10 minutes at 95 °C followed by 40 cycles of 15 seconds at 95 °C and 1 minute at 60 °C. Each sample was run as triplicate.

The ΔΔCt method was used to compare relative fold changes between samples and control. T-test was used to assess the p-Value, considering data significant when p < 0.05. The genes for qRT-PCR were randomly selected among the discussed genes, choosing those transcripts falling into different biological classes.

### Western Blot

Thirty micrograms of proteins from the 3 controls and the 3 patients pools testis biopsies were separated on SDS-PAGE and subsequently transferred to nitrocellulose sheets using a semidry blotting apparatus. Sheets were saturated for 120 min at rate temperature in blocking buffer (1xTBS, 5% milk, 0.1% Tween-20), then incubated overnight at 4 °C in blocking buffer containing primary antibodies to JUN (3 μg/mL), BCL2 (1:50), SPA17 (1:500), and β-actin (1:500). Primary antibodies to JUN and BCL2 were purchased from ThermoFisher Scientific (Waltham, MA, USA); primary antibodies to SPA17 and β-actin were purchased from Abcam (Cambridge, UK). After four washes in TBS containing 0.1% Tween-20, samples were incubated for 60 min at room temperature with peroxidase-conjugated secondary antibody diluted 1:1000 in 1x TBS, 2,5% milk, 0.1% Tween-20. Bands were visualized and quantized by the ECL method with Alliance 2.7 (UVItec Limited, Cambridge, UK). Protein levels were corrected against β-actin.

## Additional Information

**How to cite this article**: D’Aurora, M. *et al*. Testis Transcriptome Modulation in Klinefelter Patients with Hypospermatogenesis. *Sci. Rep.* **7**, 45729; doi: 10.1038/srep45729 (2017).

**Publisher's note:** Springer Nature remains neutral with regard to jurisdictional claims in published maps and institutional affiliations.

## Supplementary Material

Supplementary Files

## Figures and Tables

**Table 1:** Main clinical characteristics of Klinefelter cases and controls.

	KS subjects (n = 3)	Controls (n = 3)
Karyotype	47,XXY	46,XY
Age (yr)	29.3 ± 3.2	29.1 ± 4.4
Mean testicular volume (mL)	2.2 ± 0.7^*^	17.8 ± 2.2
Testicular histology	Hypospermatogenesis	Normal spermatogenesis (obstructive)
FSH (IU/L) (r.v. 1–8)	28.4 ± 5.7^#^	2.9 ± 0.4
LH (IU/L) (r.v. 1–9)	18.7 ± 2.7^*^	3.4 ± 0.5
Total testosterone (nmol/L) (r.v. 10–29)	10.2 ± 1.9^#^	20.3 ± 3.8

Data are expressed as mean ± standard deviation of the mean. *P < 0.01 vs controls. ^#^P < 0.05 vs controls.

## References

[b1] Aksglæde L. . Natural history of seminiferous tubule degeneration in Klinefelter syndrome. Hum. Reprod. Update. 12, 39–48 (2006). 16172111 10.1093/humupd/dmi039

[b2] Stuppia L. . A quarter of men with idiopathic oligo-azoospermia display chromosomal abnormalities and microdeletions of different types in interval 6 of Yq11. Hum. Genet. 102, 566–570 (1998). 9654206 10.1007/s004390050741

[b3] Maiburg M., Repping S. & Giltay J. The genetic origin of Klinefelter syndrome and its effect on spermatogenesis. Fertil. Steril. 98, 253–260 (2012). 22749222 10.1016/j.fertnstert.2012.06.019

[b4] Ferlin A. . Male infertility: role of genetic background. Reprod. Biomed Online. 14, 734–745 (2007). 17579990 10.1016/s1472-6483(10)60677-3

[b5] Foresta C. . Analysis of meiosis in intratesticular germ cells from subjects affected by classic Klinefelter’s syndrome. J. Clin. Endocrinol. Metab. 84, 3807–3810 (1999). 10523034 10.1210/jcem.84.10.6029

[b6] Bergère M. . Biopsied testis cells of four 47,XXY patients: fluorescence *in-situ* hybridization and ICSI results. Hum. Reprod. 17, 32–37 (2002). 11756358 10.1093/humrep/17.1.32

[b7] Abdelmoula N. B. . Cytogenetics and fluorescence *in situ* hybridization assessment of sex-chromosome mosaicism in Klinefelter’s syndrome. Ann. Genet. 47, 163–175 (2004). 15183749 10.1016/j.anngen.2003.08.024

[b8] Aksglaede L. . 47,XXY Klinefelter syndrome: Clinical characteristics and age-specific recommendations for medical management. Am. J. Med. Genet. Part C Semin. Med. Genet. 163, 55–63 (2013). 10.1002/ajmg.c.31349 23345262

[b9] Bastida M. G. . Establishment of testicular endocrine function impairment during childhood and puberty in boys with Klinefelter syndrome. Clin. Endocrinol. (Oxf). 67, 863–870 (2007). 17645574 10.1111/j.1365-2265.2007.02977.x

[b10] Wang P. J., McCarrey J. R., Yang F. & Page D. C. An abundance of X-linked genes expressed in spermatogonia. Nat. Genet. 27, 422–426 (2001). 11279525 10.1038/86927

[b11] Khil P. P., Smirnova N. A., Romanienko P. J. & Camerini-Otero R. D. The mouse X chromosome is enriched for sex-biased genes not subject to selection by meiotic sex chromosome inactivation. Nat. Genet. 36, 642–646 (2004). 15156144 10.1038/ng1368

[b12] Madureira C. . Treatment by testicular sperm extraction and intracytoplasmic sperm injection of 65 azoospermic patients with non-mosaic Klinefelter syndrome with birth of 17 healthy children. Andrology. 2, 623–631 (2014). 24954116 10.1111/j.2047-2927.2014.00231.x

[b13] D’Aurora M. . Deregulation of sertoli and leydig cells function in patients with klinefelter syndrome as evidenced by testis transcriptome analysis. BMC Genomics. 16, 156 (2015). 25879484 10.1186/s12864-015-1356-0 PMC4362638

[b14] Zheng K., Yang F. & Wang P. J. Regulation of male fertility by X-linked genes. J. Androl. 31, 79–85 (2009). 19875494 10.2164/jandrol.109.008193 PMC2931805

[b15] Kosir R. . Novel insights into the downstream pathways and targets controlled by transcription factors crem in the testis. PLoS One. 7, 2 (2012). 10.1371/journal.pone.0031798 PMC3285179 22384077

[b16] Lardenois A. . Fhl5/Act, a CREM-binding transcriptional activator required for normal sperm maturation and morphology, is not essential for testicular gene expression. Reprod. Biol. Endocrinol. 7, 133 (2009). 19930692 10.1186/1477-7827-7-133 PMC2788571

[b17] Nagamori I., Yomogida K., Adams P. D., Sassone-Corsi P. & Nojima H. Transcription factors, cAMP-responsive element modulator (CREM) and Tisp40, act in concert in postmeiotic transcriptional regulation. J. Biol. Chem. 281, 15073–15081 (2006). 16595651 10.1074/jbc.M602051200

[b18] Savadi-Shiraz E. . Quantification of sperm specific mRNA transcripts (PRM1, PRM2, and TNP2) in teratozoospermia and normozoospermia: New correlations between mRNA content and morphology of sperm. Mol. Reprod. Dev. 82, 26–35 (2015). 25536093 10.1002/mrd.22440

[b19] Song B. . Genetic study of Hormad1 and Hormad2 with non-obstructive azoospermia patients in the male Chinese population. J. Assist. Reprod. Genet. 31, 873–879 (2014). 24803422 10.1007/s10815-014-0244-x PMC4096876

[b20] Haraguchi T., Ishikawa T., Yamaguchi K. & Fujisawa M. Cyclin and protamine as prognostic molecular marker for testicular sperm extraction in patients with azoospermia. Fertil. Steril. 91, 1424–1426 (2009). 18692784 10.1016/j.fertnstert.2008.05.072

[b21] Pirouz M., Pilarski S. & Kessel M. A. Critical Function of Mad2l2 in Primordial Germ Cell Development of Mice. PLoS Genet. 9, 8 (2013). 10.1371/journal.pgen.1003712 PMC3757036 24009519

[b22] Vrooman L. A., Oatley J. M., Griswold J. E., Hassold T. J. & Hunt P. A. Estrogenic Exposure Alters the Spermatogonial Stem Cells in the Developing Testis, Permanently Reducing Crossover Levels in the Adult. PLoS Genet. 11, 1 (2015). 10.1371/journal.pgen.1004949 PMC4304829 25615633

[b23] Ly-Huynh J. D. . Importin Alpha2-Interacting Proteins with Nuclear Roles During Mammalian Spermatogenesis. Biol. Reprod. 85, 1191–1202 (2011). 21900684 10.1095/biolreprod.111.091686

[b24] Miyamoto T. . A single nucleotide polymorphism in SPATA17 may be a genetic risk factor for Japanese patients with meiotic arrest. Asian J. Androl. 11, 623–628 (2009). 19483714 10.1038/aja.2009.30 PMC3735008

[b25] Iwanaga A. . Ablation of the scaffold protein JLP causes reduced fertility in male mice. Transgenic Res. 17, 1045–1058 (2008). 18574703 10.1007/s11248-008-9191-6

[b26] Ishishita S., Inui T., Matsuda Y., Serikawa T. & Kitada K. Infertility Associated with Meiotic Failure in the tremor Rat (tm/tm) is Caused by the Deletion of Spermatogenesis Associated 22. Exp. Anim. 62, 219–227 (2013). 23903057 10.1538/expanim.62.219 PMC4160939

[b27] Lin Y. H. . The expression level of septin12 is critical for spermiogenesis. Am. J. Pathol. 174, 1857–1868 (2009). 19359518 10.2353/ajpath.2009.080955 PMC2671274

[b28] Nakamura T. . Oligo-astheno-teratozoospermia in mice lacking Cnot7, a regulator of retinoid X receptor beta. Nat. Genet. 36, 528–533 (2004). 15107851 10.1038/ng1344

[b29] Cheung-Flynn J. . Physiological role for the cochaperone FKBP52 in androgen receptor signaling. Mol. Endocrinol. 19, 1654–1966 (2005). 15831525 10.1210/me.2005-0071

[b30] Feng S., Bogatcheva N. V., Kamat A. A., Truong A. & Agoulnik A. I. Endocrine effects of relaxin overexpression in mice. Endocrinology. 147, 407–414 (2006). 16223865 10.1210/en.2005-0626

[b31] Zhou H. . Taf7l cooperates with Trf2 to regulate spermiogenesis. Proc. Natl. Acad. Sci. USA 110, 16886–16891 (2013). 24082143 10.1073/pnas.1317034110 PMC3801064

[b32] Moretti E., Scapigliati G., Pascarelli N. A., Baccetti B. & Collodel G. Localization of AKAP4 and tubulin proteins in sperm with reduced motility. Asian J. Androl. 9, 641–649 (2007). 17712481 10.1111/j.1745-7262.2007.00267.x

[b33] Berruti G. & Paiardi C. The dynamic of the apical ectoplasmic specialization between spermatids and sertoli cells: The case of the small GTPase Rap1. BioMed Res. Int. 2014 (2014). 10.1155/2014/635979 PMC3955676 24719879

[b34] Goldberg E., Eddy E. M., Duan C. & Odet F. LDHC: the ultimate testis-specific gene. J. Androl. 31, 86–94 (2010). 19875487 10.2164/jandrol.109.008367 PMC2915756

[b35] Yang Z., Yoshioka H. & McCarrey J. R. Sequence-specific promoter elements regulate temporal-specific changes in chromatin required for testis-specific activation of the Pgk2 gene. Reproduction. 146, 501–516 (2013). 24000349 10.1530/REP-13-0311 PMC3891403

[b36] Dorosh A. . Expression analysis of MND1/GAJ, SPATA22, GAPDHS and ACR genes in testicular biopsies from non-obstructive azoospermia (NOA) patients. Reprod. Biol. Endocrinol. 11, 42 (2013). 23675907 10.1186/1477-7827-11-42 PMC3664614

[b37] O’Donnell L. & O’Bryan M. K. Microtubules and spermatogenesis. Semin. Cell. Dev. Biol. 30, 45–54 (2014). 24440897 10.1016/j.semcdb.2014.01.003

[b38] Lie P. P. Y., Cheng C. Y. & Mruk D. D. Signalling pathways regulating the blood-testis barrier. Int. J. Biochem. Cell Biol. 45, 621–625 (2013). 23262290 10.1016/j.biocel.2012.12.009 PMC3632505

[b39] Pelletier R. M. The blood-testis barrier: The junctional permeability, the proteins and the lipids. Prog. Histochem. Cytochem. 46, 49–127 (2011). 21705043 10.1016/j.proghi.2011.05.001

[b40] Griswold M. D., Morales C. & Sylvester S. R. Molecular biology of the Sertoli cell. Oxf. Rev. Reprod. Biol. 10, 124–161 (1988). 3072501

[b41] Martins A. D. . Control of Sertoli cell metabolism by sex steroid hormones is mediated through modulation in glycolysis-related transporters and enzymes. Cell Tissue Res. 354, 861–868 (2013). 24057877 10.1007/s00441-013-1722-7

[b42] Hedger M. P. & Winnall W. R. Regulation of activin and inhibin in the adult testis and the evidence for functional roles in spermatogenesis and immunoregulation. Mol. Cell. Endocrinol. 359, 30–42 (2012). 21964464 10.1016/j.mce.2011.09.031

[b43] Yao B., Rieanrakwong D. & Kawaminami M. Testicular annexin A5 expression augmented by experimental cryptorchidism and could affect germ cell apoptosis in rats. Urology. 73, 1412–1416 (2009). 19376566 10.1016/j.urology.2008.11.021

[b44] Nóbrega R. H. . Fsh Stimulates Spermatogonial Proliferation and Differentiation in Zebrafish via Igf3. Endocrinology. 156, 3804–3817 (2015). 26207345 10.1210/en.2015-1157

[b45] Kaftanovskaya E. M., Lopez C., Ferguson L., Myhr C. & Agoulnik A. I. Genetic ablation of androgen receptor signaling in fetal Leydig cell lineage affects Leydig cell functions in adult testis. FASEB J. 29, 2327–2337 (2015). 25713029 10.1096/fj.14-263632 PMC6137449

[b46] Jaiswal D., Trivedi S., Agrawal N. K. & Singh K. Dysregulation of apoptotic pathway candidate genes and proteins in infertile azoospermia patients. Fertil. Steril. 104, 736–743.e6 (2015). 26056927 10.1016/j.fertnstert.2015.05.029

[b47] Xu B. . Testicular lumicrine factors regulate ERK, STAT, and NFKB pathways in the initial segment of the rat epididymis to prevent apoptosis. Biol. Reprod. 84, 1282–1291 (2011). 21311037 10.1095/biolreprod.110.090324 PMC3099589

[b48] Frungieri M. B., Calandra R. S., Mayerhofer A. & Matzkin M. E. Cyclooxygenase and prostaglandins in somatic cell populations of the testis. Reproduction. 149, R169–R180 (2015). 25504871 10.1530/REP-14-0392

[b49] Di-Luoffo M., Daems C., Bergeron F. & Tremblay J. J. Novel Targets for the Transcription Factors MEF2 in MA-10 Leydig Cells. Biol. Reprod. 93, 9 (2015). 26019261 10.1095/biolreprod.114.127761 PMC4706312

[b50] Jin Y., Chen G. & Fu Z. Effects of TBEP on the induction of oxidative stress and endocrine disruption in Tm3 Leydig cells. Environ. Toxicol. 31, 1276–1286 (2016). 25808963 10.1002/tox.22137

[b51] Ma Y. . Aberrant gene expression profiles in pluripotent stem cells induced from fibroblasts of a Klinefelter syndrome patient. J. Biol. Chem. 287, 38970–38979 (2012). 23019320 10.1074/jbc.M112.380204 PMC3493938

[b52] Zitzmann M. . Gene expression patterns in relation to the clinical phenotype in klinefelter syndrome. J. Clin. Endocrinol. Metab. 100, E518–523 (2015). 25532039 10.1210/jc.2014-2780

[b53] Zhuang X. . Integrated miRNA and mRNA expression profiling to identify mRNA targets of dysregulated miRNAs in non-obstructive azoospermia. Sci. Rep. 5, 7922 (2015). 25628250 10.1038/srep07922 PMC4310093

[b54] Huang J. . Global transcriptome analysis of peripheral blood identifies the most significantly down-regulated genes associated with metabolism regulation in Klinefelter syndrome. Mol. Reprod. Dev. 82, 17–25 (2015). 25581374 10.1002/mrd.22438

[b55] Gatta V. . Testis transcriptome analysis in male infertility: new insight on the pathogenesis of oligo-azoospermia in cases with and without AZFc microdeletion. BMC Genomics. 11, 401 (2010). 20576090 10.1186/1471-2164-11-401 PMC2996929

[b56] Storey J. D. & Tibshirani R. Statistical methods for identifying differentially expressed genes in DNA microarrays. Methods Mol. Biol. 224, 149–157 (2003). 12710672 10.1385/1-59259-364-X:149

