# Supplementary material for: Testis Transcriptome Modulation in Klinefelter Patients with Hypospermatogenesis
Source: Sci Rep. 2017 Mar 31;7:45729. doi: 10.1038/srep45729 (PMC5374630; doi:10.1038/srep45729)
Supplement: Supplementary Files [file srep45729-s1.pdf]

Testis Transcriptome Modulation in Klinefelter Patients with Hypospermatogenesis

Marco D'Aurora, Alberto Ferlin, Andrea Garolla, Sara Franchi , Laura D'Onofrio, Oriana Trubiani, Giandomenico Palka, Carlo Foresta, Liborio Stuppia, Valentina Gatta

Supplementary Table S1. Down-regulated Genes in Cluster A

ID	Symbol	Entrez Gene Name	Location	Type(s)
NM_080282.3	ABCA10	ATP binding cassette subfamily A member 10	Other	transporter
NM_080284.2	ABCA6	ATP binding cassette subfamily A member 6	Plasma Membrane	transporter
NM_021160.2	ABHD16A	abhydrolase domain containing 16A	Other	other
NM_080622.3	ABHD16B	abhydrolase domain containing 16B	Other	other
NM_172027.2	ABTB1	ankyrin repeat and BTB (POZ) domain containing 1	Cytoplasm	translation regulator
NM_001164815.1	ACIN1	apoptotic chromatin condensation inducer 1	Nucleus	enzyme
NM_018473.3	ACOT13	acyl-CoA thioesterase 13	Cytoplasm	enzyme
NM_016188.4	ACTL6B	actin like 6B	Nucleus	other
NM_006687.2	ACTL7A	actin like 7A	Nucleus	other
NM_006686.3	ACTL7B	actin like 7B	Cytoplasm	other
NM_178525.3	ACTL9	actin like 9	Other	other
NM_005736.3	ACTR1A	ARP1 actin-related protein 1 homolog A, centractin alpha (yeast)	Cytoplasm	other
NR_001569.2	ADAM3A	ADAM metallopeptidase domain 3A (pseudogene)	Other	other
NM_014921.4	ADGRL1	adhesion G protein-coupled receptor L1	Plasma Membrane	G-protein coupled receptor
NM_000677.3	ADORA3	adenosine A3 receptor	Plasma Membrane	G-protein coupled receptor
NM_031946.4	AGAP3	ArfGAP with GTPase domain, ankyrin repeat and PH domain 3	Nucleus	transcription regulator
NM_018046.3	AGGF1	angiogenic factor with G-patch and FHA domains 1	Cytoplasm	other
NM_001161766.1	AHCY	adenosylhomocysteinase	Cytoplasm	enzyme
NM_152392.3	AHSA2	AHA1, activator of heat shock 90kDa protein ATPase homolog 2 (yeast)	Other	other
NM_018836.3	AJAP1	adherens junctions associated protein 1	Plasma Membrane	other
NM_032876.4	AJUBA	ajuba LIM protein	Nucleus	transcription regulator
NM_001625.2	AK2	adenylate kinase 2	Cytoplasm	kinase

NM_139289.1	AKAP4	A-kinase anchoring protein 4	Cytoplasm	other
NM_001353.5	AKR1C1/AKR1C2	aldo-keto reductase family 1, member C2	Cytoplasm	enzyme
NM_001012398.1	AKTIP	AKT interacting protein	Cytoplasm	other
NM_003888.2	ALDH1A2	aldehyde dehydrogenase 1 family member A2	Cytoplasm	enzyme
NM_019109.4	ALG1	ALG1, chitobiosyldiphosphodolichol beta-mannosyltransferase	Cytoplasm	enzyme
NM_024079.4	ALG8	ALG8, alpha-1,3-glucosyltransferase	Cytoplasm	enzyme
NM_032306.2	ALKBH7	alkB homolog 7	Cytoplasm	other
NM_138775.2	ALKBH8	alkB homolog 8, tRNA methyltransferase	Cytoplasm	enzyme
NM_052947.3	ALPK2	alpha kinase 2	Nucleus	kinase
NM_001167595.1	AMACR	alpha-methylacyl-CoA racemase	Cytoplasm	enzyme
NM_001167595.1	AMACR	alpha-methylacyl-CoA racemase	Cytoplasm	enzyme
NM_016627.4	AMZ2	archaelysin family metallopeptidase 2	Other	other
NM_032139.2	ANKRD27	ankyrin repeat domain 27	Cytoplasm	other
NM_001039888.2	ANKRD34A	ankyrin repeat domain 34A	Cytoplasm	other
NM_173595.3	ANKRD52	ankyrin repeat domain 52	Nucleus	transcription regulator
NM_181670.2	ANKS1B	ankyrin repeat and sterile alpha motif domain containing 1B	Nucleus	other
NM_001145646.1	APH1B	aph-1 homolog B, gamma secretase subunit	Other	peptidase
NM_032389.3	ARFGAP2	ADP ribosylation factor GTPase activating protein 2	Nucleus	other
NM_001010000.1	ARHGAP28	Rho GTPase activating protein 28	Cytoplasm	other
NM_004491.4	ARHGAP35	Rho GTPase activating protein 35	Nucleus	transcription regulator
NM_032496.2	ARHGAP9	Rho GTPase activating protein 9	Cytoplasm	other
NM_006465.2	ARID3B	AT-rich interaction domain 3B	Nucleus	transcription regulator
NM_025047.2	ARL14	ADP ribosylation factor like GTPase 14	Other	other
NM_001667.2	ARL2	ADP ribosylation factor like GTPase 2	Cytoplasm	enzyme
NM_025080.3	ASRGL1	asparaginase like 1	Cytoplasm	enzyme
NM_014109.3	ATAD2	ATPase family, AAA domain containing 2	Nucleus	enzyme
NM_001130059.1	ATF7	activating transcription factor 7	Nucleus	transcription regulator
NM_022488.3	ATG3	autophagy related 3	Cytoplasm	enzyme
NM_015915.4	ATL1	atlastin GTPase 1	Cytoplasm	enzyme
NM_004231.2	ATP6V1F	ATPase, H+ transporting, lysosomal 14kDa, V1 subunit F	Cytoplasm	enzyme
NM_004231.2	ATP6V1F	ATPase, H+ transporting, lysosomal 14kDa, V1 subunit F	Cytoplasm	enzyme
NM_016311.3	ATPIF1	ATPase inhibitory factor 1	Cytoplasm	other
NM_153340.4	ATXN7L2	ataxin 7-like 2	Other	other
NM_000490.4	AVP	arginine vasopressin	Other	other
NM_001005417.1	B4GALT2	UDP-Gal:betaGlcNAc beta 1,4-galactosyltransferase, polypeptide 2	Cytoplasm	enzyme
NM_203314.2	BDH1	3-hydroxybutyrate dehydrogenase, type 1	Cytoplasm	enzyme
NR_026760.1	BPIFA4P	BPI fold containing family A member 4, pseudogene	Extracellular Space	other
NM_005104.3	BRD2	bromodomain containing 2	Nucleus	kinase
NM_033271.2	BTBD6	BTB (POZ) domain containing 6	Other	other
NM_152587.3	C11orf65	chromosome 11 open reading frame 65	Other	other

NM_001037225.1	C11orf85	chromosome 11 open reading frame 85	Nucleus	other
NM_021640.3	C12orf10	chromosome 12 open reading frame 10	Nucleus	other
NM_015492.4	C15orf39	chromosome 15 open reading frame 39	Cytoplasm	other
NM_001012984.2	C16orf86	chromosome 16 open reading frame 86	Other	other
NM_001145350.1	C20orf173	chromosome 20 open reading frame 173	Other	other
NR_026933.1	C20orf173	chromosome 20 open reading frame 173	Other	other
NM_024059.2	C20orf195	chromosome 20 open reading frame 195	Cytoplasm	other
NM_178456.2	C20orf85	chromosome 20 open reading frame 85	Other	other
NM_206895.1	C2orf82	chromosome 2 open reading frame 82	Cytoplasm	other
NM_032149.2	C4orf17	chromosome 4 open reading frame 17	Other	other
NM_152770.1	C4orf22	chromosome 4 open reading frame 22	Other	other
NM_023073.3	C5orf42	chromosome 5 open reading frame 42	Other	other
NM_001102609.1	C5orf58	chromosome 5 open reading frame 58	Other	other
NM_001004323.1	C7orf61	chromosome 7 open reading frame 61	Nucleus	other
NM_144654.2	C9orf116	chromosome 9 open reading frame 116	Other	other
NM_001010940.1	C9orf135	chromosome 9 open reading frame 135	Other	other
NM_152786.1	C9orf43	chromosome 9 open reading frame 43	Other	other
NM_033122.3	CABS1	calcium-binding protein, spermatid-specific 1	Extracellular Space	other
NM_138643.1	CABYR	calcium binding tyrosine-(Y)-phosphorylation regulated	Cytoplasm	other
NM_005184.2	CALM1 (includes others)	calmodulin 1 (phosphorylase kinase, delta)	Cytoplasm	other
NM_138705.2	CALML6	calmodulin like 6	Cytoplasm	other
NM_005632.2	CAPN15	calpain 15	Other	peptidase
NM_004930.3	CAPZB	capping protein (actin filament) muscle Z-line, beta	Cytoplasm	other
NM_001042476.1	CARHSP1	calcium regulated heat stable protein 1	Cytoplasm	other
NM_001080522.2	CC2D2A	coiled-coil and C2 domain containing 2A	Cytoplasm	other
NM_022742.3	CCDC136	coiled-coil domain containing 136	Cytoplasm	other
NM_001144983.1	CCDC169	coiled-coil domain containing 169	Other	other
NM_001085447.1	CCDC173	coiled-coil domain containing 173	Other	other
NM_018017.2	CCDC186	coiled-coil domain containing 186	Other	other
NM_017950.2	CCDC40	coiled-coil domain containing 40	Cytoplasm	other
NM_144681.2	CCDC42	coiled-coil domain containing 42	Other	other
NM_016053.2	CCDC53	coiled-coil domain containing 53	Cytoplasm	other
NM_198082.2	CCDC57	coiled-coil domain containing 57	Other	other
NM_001080402.1	CCDC61	coiled-coil domain containing 61	Cytoplasm	other
NM_021825.3	CCDC90B	coiled-coil domain containing 90B	Cytoplasm	other
NM_003914.3	CCNA1	cyclin A1	Nucleus	other
NM_004701.2	CCNB2	cyclin B2	Cytoplasm	other
NM_024565.5	CCNJL	cyclin J like	Other	other
NM_021147.3	CCNO	cyclin O	Nucleus	enzyme
NM_181698.2	CCNY	cyclin Y	Nucleus	other
NM_007053.2	CD160	CD160 molecule	Plasma Membrane	transmembrane receptor

NM_174941.4	CD163L1	CD163 molecule like 1	Plasma Membrane	transmembrane receptor
NM_016579.3	CD320	CD320 molecule	Plasma Membrane	other
NM_007065.3	CDC37	cell division cycle 37	Cytoplasm	other
NM_080668.3	CDCA5	cell division cycle associated 5	Cytoplasm	other
NM_176096.1	CDK5RAP3	CDK5 regulatory subunit associated protein 3	Nucleus	other
NM_001799.3	CDK7	cyclin-dependent kinase 7	Nucleus	kinase
NM_005192.3	CDKN3	cyclin-dependent kinase inhibitor 3	Cytoplasm	phosphatase
NM_001029954.2	CDNF	cerebral dopamine neurotrophic factor	Other	other
NM_001802.1	CDR2	cerebellar degeneration related protein 2	Cytoplasm	other
NM_017548.4	CDV3	CDV3 homolog (mouse)	Cytoplasm	other
NM_024322.1	CENPO	centromere protein O	Nucleus	other
NM_024629.3	CENPU	centromere protein U	Nucleus	other
NM_014810.4	CEP350	centrosomal protein 350kDa	Cytoplasm	other
NM_018131.4	CEP55	centrosomal protein 55kDa	Cytoplasm	other
NM_001098802.1	CEP78	centrosomal protein 78kDa	Cytoplasm	other
NR_003276.2	CES1P1	carboxylesterase 1 pseudogene 1	Cytoplasm	enzyme
NM_001013625.2	CFAP126	cilia and flagella associated protein 126	Other	other
NM_207417.1	CFAP77	cilia and flagella associated protein 77	Other	other
NM_001127183.1	CFLAR	CASP8 and FADD like apoptosis regulator	Cytoplasm	other
NM_001008708.2	CHAC2	ChaC, cation transport regulator homolog 2 (E. coli)	Cytoplasm	other
NM_001819.2	CHGB	chromogranin B	Extracellular Space	other
NM_004198.2	CHRNA6	cholinergic receptor, nicotinic alpha 6	Plasma Membrane	transmembrane receptor
NM_001040138.1	CKLF	chemokine-like factor	Extracellular Space	cytokine
NM_001099431.1	CLEC1B	C-type lectin domain family 1 member B	Plasma Membrane	transmembrane receptor
NM_207345.2	CLEC9A	C-type lectin domain family 9 member A	Plasma Membrane	other
NM_004669.2	CLIC3	chloride intracellular channel 3	Nucleus	ion channel
NM_003993.2	CLK2	CDC like kinase 2	Nucleus	kinase
NM_006831.2	CLP1	cleavage and polyadenylation factor I subunit 1	Nucleus	other
NM_030813.3	CLPB	ClpB homolog, mitochondrial AAA ATPase chaperonin	Nucleus	transcription regulator
NM_014718.3	CLSTN3	calsyntenin 3	Plasma Membrane	other
NM_020188.3	CMC2	C-x(9)-C motif containing 2	Cytoplasm	other
NM_052999.3	CMTM1	CKLF-like MARVEL transmembrane domain containing 1	Other	other
NM_144673.2	CMTM2	CKLF-like MARVEL transmembrane domain containing 2	Extracellular Space	cytokine
NM_206999.1	CNOT1	CCR4-NOT transcription complex subunit 1	Cytoplasm	other
NM_054026.2	CNOT7	CCR4-NOT transcription complex subunit 7	Nucleus	transcription regulator
NM_175607.1	CNTN4	contactin 4	Plasma Membrane	enzyme
NM_080801.3	COL13A1	collagen, type XIII, alpha 1	Plasma Membrane	other
NM_152516.2	COMMD1	copper metabolism domain containing 1	Nucleus	transporter
NM_018405.3	COPRS	coordinator of PRMT5, differentiation stimulator	Nucleus	other

NM_001030005.2	CPLX3	complexin 3	Nucleus	transporter
NM_016207.2	CPSF3	cleavage and polyadenylation specific factor 3	Nucleus	enzyme
NM_001145135.1	CPT1B	carnitine palmitoyltransferase 1B	Cytoplasm	enzyme
NM_019609.4	CPXM1	carboxypeptidase X (M14 family), member 1	Extracellular Space	peptidase
NM_001878.2	CRABP2	cellular retinoic acid binding protein 2	Cytoplasm	transporter
NM_024324.3	CRELD2	cysteine rich with EGF-like domains 2	Other	other
NM_182769.1	CREM	cAMP responsive element modulator	Nucleus	transcription regulator
NM_182769.1	CREM	cAMP responsive element modulator	Nucleus	transcription regulator
NM_153605.2	CRYBG3	crystallin beta-gamma domain containing 3	Other	other
NM_145858.2	CRYZL1	crystallin zeta like 1	Cytoplasm	enzyme
NM_022579.1	CSHL1	chorionic somatomammotropin hormone like 1	Nucleus	transcription regulator
NM_022048.3	CSNK1G1	casein kinase 1, gamma 1	Cytoplasm	kinase
NM_001319.6	CSNK1G2	casein kinase 1, gamma 2	Cytoplasm	kinase
NM_030809.1	CSRNP2	cysteine-serine-rich nuclear protein 2	Nucleus	transcription regulator
NR_001279.2	CST13P	cystatin 13, pseudogene	Other	other
NM_005492.2	CST8	cystatin 8	Extracellular Space	other
NM_001911.2	CTSG	cathepsin G	Cytoplasm	peptidase
NM_003591.2	CUL2	cullin 2	Nucleus	enzyme
NM_199168.3	CXCL12	chemokine (C-X-C motif) ligand 12	Extracellular Space	cytokine
NM_022059.2	CXCL16	chemokine (C-X-C motif) ligand 16	Extracellular Space	cytokine
NM_016229.3	CYB5R2	cytochrome b5 reductase 2	Cytoplasm	enzyme
NM_001916.3	CYC1	cytochrome c-1	Cytoplasm	enzyme
NM_000773.3	CYP2E1	cytochrome P450 family 2 subfamily E member 1	Cytoplasm	enzyme
NM_004227.3	CYTH3	cytohesin 3	Cytoplasm	other
NM_138709.1	DAB2IP	DAB2 interacting protein	Plasma Membrane	other
NM_178821.1	DAW1	dynein assembly factor with WDR repeat domains 1	Other	other
NR_027642.1	DCAF13P3	DDB1 and CUL4 associated factor 13 pseudogene 3	Other	other
NM_033403.1	DCLK3	doublecortin like kinase 3	Cytoplasm	kinase
NM_020414.3	DDX24	DEAD-box helicase 24	Nucleus	enzyme
NM_013264.3	DDX25	DEAD-box helicase 25	Nucleus	enzyme
NM_001925.1	DEFA4	defensin alpha 4	Extracellular Space	other
NM_001077242.1	DEPDC7	DEP domain containing 7	Cytoplasm	other
NM_012079.4	DGAT1	diacylglycerol O-acyltransferase 1	Cytoplasm	enzyme
NM_014419.3	DKKL1	dickkopf-like 1	Extracellular Space	other
NM_004405.3	DLX2	distal-less homeobox 2	Nucleus	transcription regulator
NM_005528.3	DNAJC4	DnaJ heat shock protein family (Hsp40) member C4	Cytoplasm	other
NR_029431.1	DNAJC7	DnaJ heat shock protein family (Hsp40) member C7	Cytoplasm	other
NM_014705.3	DOCK4	dedicator of cytokinesis 4	Plasma	other

			Membrane	
NM_032482.2	DOT1L	DOT1-like histone H3K79 methyltransferase	Nucleus	phosphatase
NR_003551.1	DPY19L2P4	DPY19L2 pseudogene 4	Other	other
NR_026838.1	DSCR8	Down syndrome critical region 8	Other	other
NM_183360.1	DTNB	dystrobrevin beta	Plasma Membrane	other
NM_183040.1	DTNBP1	dystrobrevin binding protein 1	Plasma Membrane	other
NM_004416.2	DTX1	deltex 1, E3 ubiquitin ligase	Nucleus	transcription regulator
NM_130897.1	DYNLRB2	dynein, light chain, roadblock-type 2	Cytoplasm	other
NM_006483.1	DYRK1B	dual specificity tyrosine-(Y)-phosphorylation regulated kinase 1B	Nucleus	kinase
NM_001950.3	E2F4	E2F transcription factor 4, p107/p130-binding	Nucleus	transcription regulator
NM_001077693.2	ECSCR	endothelial cell surface expressed chemotaxis and apoptosis regulator	Other	other
NM_001959.3	EEF1B2	eukaryotic translation elongation factor 1 beta 2	Cytoplasm	translation regulator
NR_027068.1	EFCAB10	EF-hand calcium binding domain 10	Other	other
NM_024757.4	EHMT1	euchromatic histone-lysine N-methyltransferase 1	Nucleus	transcription regulator
NM_003751.3	EIF3B	eukaryotic translation initiation factor 3 subunit B	Cytoplasm	translation regulator
NM_001099661.1	EIF3CL	eukaryotic translation initiation factor 3 subunit C-like	Other	other
NM_002212.2	EIF6	eukaryotic translation initiation factor 6	Cytoplasm	translation regulator
NM_004097.2	EMX1	empty spiracles homeobox 1	Nucleus	transcription regulator
NM_001184937.1	EPB41L5	erythrocyte membrane protein band 4.1 like 5	Plasma Membrane	other
NM_004447.5	EPS8	epidermal growth factor receptor pathway substrate 8	Plasma Membrane	peptidase
NM_007277.4	EXOC3	exocyst complex component 3	Plasma Membrane	transporter
NM_019037.2	EXOSC4	exosome component 4	Nucleus	enzyme
NM_004456.3	EZH2	enhancer of zeste 2 polycomb repressive complex 2 subunit	Nucleus	transcription regulator
NR_003698.1	FAHD2CP	fumarylacetoacetate hydrolase domain containing 2C, pseudogene	Other	other
NM_181709.3	FAM101A/ZNF664-FAM101A	family with sequence similarity 101 member A	Other	transporter
NM_173698.2	FAM133A	family with sequence similarity 133 member A	Other	other
NM_001001710.1	FAM166A	family with sequence similarity 166 member A	Nucleus	other
NM_015091.2	FAM179B	family with sequence similarity 179 member B	Extracellular Space	other
NR_024019.1	FAM193B	family with sequence similarity 193 member B	Nucleus	other
NM_001013646.2	FAM209B	family with sequence similarity 209 member B	Nucleus	other
NR_027751.1	FAM20A	family with sequence similarity 20 member A	Extracellular Space	other
NM_001077498.1	FAM222B	family with sequence similarity 222 member B	Nucleus	other
NM_198488.3	FAM83H	family with sequence similarity 83 member H	Other	other
NM_001171186.1	FAM9A	family with sequence similarity 9 member A	Nucleus	other
NM_145235.3	FANK1	fibronectin type III and ankyrin repeat domains 1	Nucleus	transcription

				regulator
NM_001024216.1	FBLIM1	filamin binding LIM protein 1	Plasma Membrane	other
NM_001105079.1	FBRS	fibrosin	Extracellular Space	cytokine
NM_024963.4	FBXL18	F-box and leucine-rich repeat protein 18	Other	enzyme
NM_152676.2	FBXO15	F-box protein 15	Other	transcription regulator
NM_012173.3	FBXO25	F-box protein 25	Nucleus	enzyme
NM_031282.2	FCRL4	Fc receptor-like 4	Cytoplasm	other
NM_194429.1	FGFR1OP	FGFR1 oncogene partner	Cytoplasm	kinase
NM_001166243.1	FHIT	fragile histidine triad	Cytoplasm	enzyme
NM_020482.4	FHL5	four and a half LIM domains 5	Nucleus	transcription regulator
NM_015687.2	FILIP1	filamin A interacting protein 1	Cytoplasm	other
NM_001134937.1	FIP1L1	factor interacting with PAPOLA and CPSF1	Nucleus	other
NM_002014.3	FKBP4	FK506 binding protein 4	Nucleus	enzyme
NM_152578.2	FMR1NB	fragile X mental retardation 1 neighbor	Nucleus	other
NM_012183.2	FOXD3	forkhead box D3	Nucleus	transcription regulator
NM_001454.3	FOXJ1	forkhead box J1	Nucleus	transcription regulator
NM_005197.3	FOXN3	forkhead box N3	Nucleus	transcription regulator
NM_001462.3	FPR2	formyl peptide receptor 2	Plasma Membrane	G-protein coupled receptor
NM_001013439.1	FXR1	fragile X mental retardation, autosomal homolog 1	Cytoplasm	other
NM_001127621.1	GALE	UDP-galactose-4-epimerase	Cytoplasm	enzyme
NM_014364.4	GAPDHS	glyceraldehyde-3-phosphate dehydrogenase, spermatogenic	Cytoplasm	enzyme
NM_182828.2	GDF7	growth differentiation factor 7	Extracellular Space	growth factor
NM_030792.6	GDPD5	glycerophosphodiester phosphodiesterase domain containing 5	Plasma Membrane	enzyme
NM_015721.2	GEMIN4	gem nuclear organelle associated protein 4	Nucleus	other
NR_024573.1	GHDC	GH3 domain containing	Cytoplasm	other
NM_022574.4	GIGYF1	GRB10 interacting GYF protein 1	Extracellular Space	other
NM_153236.3	GIMAP7	GTPase, IMAP family member 7	Cytoplasm	enzyme
NM_014030.3	GIT1	G protein-coupled receptor kinase interacting ArfGAP 1	Nucleus	kinase
NM_033214.2	GK2	glycerol kinase 2	Cytoplasm	kinase
NM_033214.2	GK2	glycerol kinase 2	Cytoplasm	kinase
NM_019617.3	GKN1	gastrokine 1	Extracellular Space	growth factor
NM_005275.3	GNL1	guanine nucleotide binding protein-like 1	Nucleus	other
NM_178331.1	GNRH2	gonadotropin releasing hormone 2	Extracellular Space	other
NR_027411.1	GOLGA8CP	golgin A8 family member C, pseudogene	Other	other
NM_004871.2	GOSR1	golgi SNAP receptor complex member 1	Cytoplasm	transporter
NM_018040.2	GPATCH2	G-patch domain containing 2	Other	other
NR_026735.1	GPN1	GPN-loop GTPase 1	Nucleus	transcription regulator

NM_001080452.1	GPR108	G protein-coupled receptor 108	Plasma Membrane	G-protein coupled receptor
NM_000273.2	GPR143	G protein-coupled receptor 143	Plasma Membrane	G-protein coupled receptor
NM_199243.1	GPR150	G protein-coupled receptor 150	Plasma Membrane	G-protein coupled receptor
NM_001508.2	GPR39	G protein-coupled receptor 39	Plasma Membrane	G-protein coupled receptor
NM_198281.2	GPRIN3	GPRIN family member 3	Other	other
NM_004489.4	GPS2	G protein pathway suppressor 2	Nucleus	transcription regulator
NM_000826.3	GRIA2	glutamate receptor, ionotropic, AMPA 2	Plasma Membrane	ion channel
NM_031415.2	GSDMC	gasdermin C	Cytoplasm	other
NM_001514.5	GTF2B	general transcription factor IIB	Nucleus	transcription regulator
NM_002096.2	GTF2F1	general transcription factor IIF subunit 1	Nucleus	transcription regulator
NM_012087.3	GTF3C5	general transcription factor IIIC subunit 5	Nucleus	transcription regulator
NM_144594.2	GTSF1	gametocyte specific factor 1	Cytoplasm	other
NM_033553.2	GUCA2A	guanylate cyclase activator 2A	Extracellular Space	other
NM_004130.3	GYG1	glycogenin 1	Cytoplasm	enzyme
NM_181788.1	H1FNT	H1 histone family member N, testis specific	Nucleus	other
NM_014282.2	HABP4	hyaluronan binding protein 4	Cytoplasm	other
NM_014282.2	HABP4	hyaluronan binding protein 4	Cytoplasm	other
NM_001010915.3	HACD4	3-hydroxyacyl-CoA dehydratase 4	Cytoplasm	enzyme
NM_033647.2	HELB	helicase (DNA) B	Nucleus	enzyme
NM_022079.2	HERC4	HECT and RLD domain containing E3 ubiquitin protein ligase 4	Cytoplasm	enzyme
NM_152419.2	HGSNAT	heparan-alpha-glucosaminide N-acetyltransferase	Cytoplasm	enzyme
NM_022462.3	HIF3A	hypoxia inducible factor 3, alpha subunit	Nucleus	transcription regulator
NM_003542.3	HIST1H4C	histone cluster 1, H4c	Nucleus	other
NM_175054.2	HIST4H4	histone cluster 4, H4	Nucleus	other
NM_033496.2	HK1	hexokinase 1	Cytoplasm	kinase
NM_025130.3	HKDC1	hexokinase domain containing 1	Other	kinase
NR_024240.1	HLA-J	major histocompatibility complex, class I, J (pseudogene)	Other	other
NM_020187.2	HMCES	5-hydroxymethylcytosine (hmC) binding, ES cell-specific	Other	other
NM_016185.2	HN1	hematological and neurological expressed 1	Nucleus	other
NM_152510.2	HORMAD2	HORMA domain containing 2	Nucleus	other
NM_020386.3	HRASLS	HRAS-like suppressor	Cytoplasm	enzyme
NM_021979.3	HSPA2	heat shock protein family A (Hsp70) member 2	Cytoplasm	other
NM_001039613.1	IAH1	isoamyl acetate-hydrolyzing esterase 1 homolog	Cytoplasm	other
NM_138468.4	ICA1L	islet cell autoantigen 1 like	Other	other
NM_006546.3	IGF2BP1	insulin like growth factor 2 mRNA binding protein 1	Cytoplasm	translation regulator
NM_001002923.1	IGFL4	IGF like family member 4	Extracellular Space	other

NM_003856.2	IL1RL1	interleukin 1 receptor like 1	Plasma Membrane	transmembrane receptor
NM_000588.3	IL3	interleukin 3	Extracellular Space	cytokine
NM_172374.1	IL4I1	interleukin 4 induced 1	Cytoplasm	enzyme
NM_001128928.1	INPP1	inositol polyphosphate-1-phosphatase	Cytoplasm	phosphatase
NM_003866.2	INPP4B	inositol polyphosphate-4-phosphatase type II B	Cytoplasm	phosphatase
NM_017864.2	INTS8	integrator complex subunit 8	Nucleus	other
NM_016291.2	IP6K2	inositol hexakisphosphate kinase 2	Cytoplasm	kinase
NM_014652.3	IPO13	importin 13	Nucleus	transporter
NM_152397.2	IQCF1	IQ motif containing F1	Cytoplasm	other
NM_004136.2	IREB2	iron responsive element binding protein 2	Cytoplasm	translation regulator
NM_080826.1	ISM1	isthmin 1, angiogenesis inhibitor	Other	other
NM_002227.2	JAK1	Janus kinase 1	Cytoplasm	kinase
NM_005472.4	KCNE3	potassium channel, voltage gated subfamily E regulatory beta subunit 3	Plasma Membrane	ion channel
NM_000238.2	KCNH2	potassium channel, voltage gated eag related subfamily H, member 2	Plasma Membrane	ion channel
NM_000891.2	KCNJ2	potassium channel, inwardly rectifying subfamily J, member 2	Plasma Membrane	ion channel
NM_004823.1	KCNK6	potassium channel, two pore domain subfamily K, member 6	Plasma Membrane	ion channel
NM_007035.3	KERA	keratocan	Extracellular Space	other
NM_014773.3	KIAA0141	KIAA0141	Cytoplasm	other
NM_001099294.1	KIAA1644	KIAA1644	Other	other
NM_194313.2	KIF24	kinesin family member 24	Cytoplasm	other
NM_004521.2	KIF5B	kinesin family member 5B	Cytoplasm	other
NM_001130100.1	KIFC3	kinesin family member C3	Cytoplasm	enzyme
NM_001163023.1	KIZ	kizuna centrosomal protein	Cytoplasm	other
NM_001134775.1	KLC2	kinesin light chain 2	Cytoplasm	other
NM_138693.2	KLF14	Kruppel-like factor 14	Nucleus	other
NM_001160124.1	KLF6	Kruppel-like factor 6	Nucleus	transcription regulator
NM_057161.2	KLHDC3	kelch domain containing 3	Cytoplasm	other
NM_152366.4	KLHDC9	kelch domain containing 9	Other	other
NM_032775.2	KLHL22	kelch like family member 22	Cytoplasm	other
NM_006063.2	KLHL41	kelch like family member 41	Cytoplasm	other
NM_004917.3	KLK4	kallikrein related peptidase 4	Extracellular Space	peptidase
NM_016523.1	KLRF1	killer cell lectin like receptor F1	Plasma Membrane	transmembrane receptor
NM_001097611.1	KNCN	kinocilin	Cytoplasm	other
NM_001142762.1	KNSTRN	kinetochore-localized astrin/SPAG5 binding protein	Cytoplasm	other
NM_002266.2	KPNA2	karyopherin alpha 2 (RAG cohort 1, importin alpha 1)	Nucleus	transporter
NM_181615.1	KRTAP20-1	keratin associated protein 20-1	Other	other
NM_014238.1	KSR1	kinase suppressor of ras 1	Cytoplasm	kinase
NM_006762.2	LAPTM5	lysosomal protein transmembrane 5	Plasma Membrane	other

NM_002296.2	LBR	lamin B receptor	Nucleus	enzyme
NM_005565.3	LCP2	lymphocyte cytosolic protein 2	Cytoplasm	other
NM_033195.1	LDHAL6B	lactate dehydrogenase A-like 6B	Cytoplasm	enzyme
NM_002301.4	LDHC	lactate dehydrogenase C	Cytoplasm	enzyme
NM_001010857.1	LELP1	late cornified envelope-like proline-rich 1	Other	other
NM_020129.2	LGALS14	lectin, galactoside-binding, soluble, 14	Nucleus	other
NM_001001933.1	LHX8	LIM homeobox 8	Nucleus	transcription regulator
NM_001004317.2	LIN28B	lin-28 homolog B (C. elegans)	Cytoplasm	other
NM_194282.2	LIN54	lin-54 DREAM MuvB core complex component	Nucleus	other
NR_024347.1	LINC00304	long intergenic non-protein coding RNA 304	Other	other
NR_026761.2	LINC00467	long intergenic non-protein coding RNA 467	Other	other
NR_026761.2	LINC00467	long intergenic non-protein coding RNA 467	Other	other
NR_028326.1	LINC01001 (includes others)	long intergenic non-protein coding RNA 1347	Other	other
NM_152590.2	LMNTD1	lamin tail domain containing 1	Nucleus	other
NM_001085451.1	LNP1	leukemia NUP98 fusion partner 1	Other	other
XM_001716669.2	LOC100131347	RAD52 motif containing 1 pseudogene	Other	other
NR_024264.1	LOC145845	uncharacterized LOC145845	Other	other
NR_028335.1	LOC284009	uncharacterized LOC284009	Other	other
NR_015361.1	LOC440896	uncharacterized LOC440896	Other	other
NM_024036.4	LRFN4	leucine rich repeat and fibronectin type III domain containing 4	Plasma Membrane	other
NM_024036.4	LRFN4	leucine rich repeat and fibronectin type III domain containing 4	Plasma Membrane	other
NM_018214.4	LRRC1	leucine rich repeat containing 1	Cytoplasm	other
XM_059074.6	LRRC38	leucine rich repeat containing 38	Other	other
NM_144620.2	LRRC39	leucine rich repeat containing 39	Other	other
NM_001137551.1	LRRFIP1	leucine rich repeat (in FLII) interacting protein 1	Cytoplasm	other
NM_001105659.1	LRRIQ3	leucine-rich repeats and IQ motif containing 3	Other	other
NR_026886.1	LRTOMT	leucine rich transmembrane and O-methyltransferase domain containing	Other	enzyme
NM_001145725.1	LYAR	Ly1 antibody reactive	Plasma Membrane	other
NM_173506.4	LYPD4	LY6/PLAUR domain containing 4	Other	other
NM_001164840.1	LYRM4	LYR motif containing 4	Cytoplasm	other
NM_020408.4	LYRM4	LYR motif containing 4	Cytoplasm	other
NM_001001660.2	LYRM5	LYR motif containing 5	Cytoplasm	other
NM_005366.4	MAGEA11	MAGE family member A11	Nucleus	other
NM_006699.3	MAN1A2	mannosidase, alpha, class 1A, member 2	Cytoplasm	enzyme
NM_006609.3	MAP3K2	mitogen-activated protein kinase kinase kinase 2	Cytoplasm	kinase
NM_020152.2	MAP3K7CL	MAP3K7 C-terminal like	Other	other
NM_024871.2	MAP6D1	MAP6 domain containing 1	Cytoplasm	other
NM_002748.3	MAPK6	mitogen-activated protein kinase 6	Cytoplasm	kinase
NM_138396.4	MARCH9	membrane associated ring-CH-type finger 9	Cytoplasm	other
NM_032844.3	MASTL	microtubule associated serine/threonine kinase like	Cytoplasm	kinase
NM_138799.2	MBOAT2	membrane bound O-acyltransferase domain containing 2	Cytoplasm	enzyme

NM_001146083.1	MBOAT7	membrane bound O-acyltransferase domain containing 7	Plasma Membrane	other
NM_001011700.2	MCCD1	mitochondrial coiled-coil domain 1	Other	other
NM_014623.2	MEA1	male-enhanced antigen 1	Cytoplasm	other
NM_015889.3	MED15	mediator complex subunit 15	Nucleus	transcription regulator
NM_133640.3	MED22	mediator complex subunit 22	Cytoplasm	other
NM_015143.2	METAP1	methionyl aminopeptidase 1	Cytoplasm	peptidase
NM_001043229.1	METTL12	methyltransferase like 12	Cytoplasm	other
NM_001080510.2	METTL23	methyltransferase like 23	Other	other
NM_032230.2	METTL25	methyltransferase like 25	Extracellular Space	other
NM_019852.3	METTL3	methyltransferase like 3	Nucleus	enzyme
NM_152599.3	MFSD6L	major facilitator superfamily domain containing 6-like	Other	other
NM_013446.3	MKRN1	makorin ring finger protein 1	Other	enzyme
NM_000249.3	MLH1	mutL homolog 1	Nucleus	enzyme
NM_001009569.1	MLLT10	myeloid/lymphoid or mixed-lineage leukemia; translocated to, 10	Nucleus	transcription regulator
NM_001040001.1	MLLT4	myeloid/lymphoid or mixed-lineage leukemia; translocated to, 4	Nucleus	other
NM_002424.2	MMP8	matrix metallopeptidase 8	Extracellular Space	peptidase
NM_005515.3	MNX1	motor neuron and pancreas homeobox 1	Nucleus	transcription regulator
NR_003090.1	MOBP	myelin-associated oligodendrocyte basic protein	Cytoplasm	other
NM_006792.2	MORF4	mortality factor 4 (pseudogene)	Nucleus	transcription regulator
NM_001145450.1	MORN2	MORN repeat containing 2	Other	other
NM_173855.4	MORN3	MORN repeat containing 3	Nucleus	other
NM_004870.3	MPDU1	mannose-P-dolichol utilization defect 1	Cytoplasm	other
NM_198275.1	MPZL3	myelin protein zero-like 3	Other	other
NM_032111.2	MRPL14	mitochondrial ribosomal protein L14	Cytoplasm	other
NM_024026.4	MRPL57	mitochondrial ribosomal protein L57	Cytoplasm	other
NM_176805.1	MRPS11	mitochondrial ribosomal protein S11	Cytoplasm	other
NM_031280.3	MRPS15	mitochondrial ribosomal protein S15	Cytoplasm	other
NM_031280.3	MRPS15	mitochondrial ribosomal protein S15	Cytoplasm	other
NM_020191.2	MRPS22	mitochondrial ribosomal protein S22	Cytoplasm	other
XM_001716326.2	MRVI1-AS1	MRVI1 antisense RNA 1	Other	other
NM_148975.1	MS4A4A	membrane-spanning 4-domains subfamily A member 4A	Cytoplasm	other
NM_023945.2	MS4A5	membrane-spanning 4-domains subfamily A member 5	Other	other
NM_020998.3	MST1	macrophage stimulating 1	Extracellular Space	growth factor
NM_014342.3	MTCH2	mitochondrial carrier 2	Cytoplasm	other
NM_001039656.1	MTL5	metallothionein-like 5, testis-specific (tesmin)	Cytoplasm	other
NM_004687.4	MTMR4	myotubularin related protein 4	Cytoplasm	phosphatase
NM_002471.3	MYH6	myosin, heavy chain 6, cardiac muscle, alpha	Cytoplasm	enzyme
NM_004145.3	MYO9B	myosin IXB	Cytoplasm	enzyme
NM_181527.2	NAA20	N(alpha)-acetyltransferase 20, NatB catalytic subunit	Cytoplasm	enzyme

NM_032356.3	NAA38	N(alpha)-acetyltransferase 38, NatC auxiliary subunit	Nucleus	other
NM_024068.3	NABP2	nucleic acid binding protein 2	Nucleus	other
NM_001113201.1	NACA	nascent polypeptide-associated complex alpha subunit	Cytoplasm	transcription regulator
NM_197956.3	NAIF1	nuclear apoptosis inducing factor 1	Nucleus	other
NM_003826.2	NAPG	N-ethylmaleimide-sensitive factor attachment protein, gamma	Cytoplasm	transporter
NM_001102663.1	NBPF15 (includes others)	neuroblastoma breakpoint family member 15	Other	other
NM_001076682.2	NCAM1	neural cell adhesion molecule 1	Plasma Membrane	other
NM_014865.3	NCAPD2	non-SMC condensin I complex subunit D2	Nucleus	other
NM_174889.4	NDUFAF2	NADH:ubiquinone oxidoreductase complex assembly factor 2	Cytoplasm	enzyme
NM_182966.3	NEDD9	neural precursor cell expressed, developmentally down-regulated 9	Nucleus	other
NM_002904.5	NELFE	negative elongation factor complex member E	Nucleus	other
NR_026598.1	NENF	neudesin neurotrophic factor	Extracellular Space	growth factor
NM_022728.2	NEUROD6	neuronal differentiation 6	Nucleus	transcription regulator
NM_002507.3	NGFR	nerve growth factor receptor	Plasma Membrane	transmembrane receptor
NM_024894.2	NOL10	nucleolar protein 10	Nucleus	other
NM_022917.4	NOL6	nucleolar protein 6	Nucleus	other
NM_001130102.1	NR1H3	nuclear receptor subfamily 1 group H member 3	Nucleus	ligand-dependent nuclear receptor
NM_001101662.1	NRDC	nardilysin convertase	Cytoplasm	peptidase
NM_001040110.1	NRF1	nuclear respiratory factor 1	Nucleus	transcription regulator
NM_022455.4	NSD1	nuclear receptor binding SET domain protein 1	Nucleus	transcription regulator
NM_145080.3	NSMCE1	NSE1 homolog, SMC5-SMC6 complex component	Nucleus	transporter
NM_033253.2	NT5C1B	5'-nucleotidase, cytosolic IB	Cytoplasm	phosphatase
NM_152729.2	NT5DC1	5'-nucleotidase domain containing 1	Other	other
NM_173474.2	NTAN1	N-terminal asparagine amidase	Nucleus	enzyme
NM_006179.4	NTF4	neurotrophin 4	Extracellular Space	growth factor
NM_014064.2	NTMT1	N-terminal Xaa-Pro-Lys N-methyltransferase 1	Nucleus	enzyme
NM_005085.2	NUP214	nucleoporin 214kDa	Nucleus	transporter
NM_007342.2	NUPL2	nucleoporin like 2	Nucleus	transporter
NM_001145712.1	NUPR2	nuclear protein 2, transcriptional regulator	Nucleus	other
NM_001134939.1	OAZ3	ornithine decarboxylase antizyme 3	Cytoplasm	transporter
NM_138983.2	OLIG1	oligodendrocyte transcription factor 1	Nucleus	transcription regulator
NM_000912.3	OPRK1	opioid receptor, kappa 1	Plasma Membrane	G-protein coupled receptor
NM_001004451.1	OR1J1	olfactory receptor family 1 subfamily J member 1	Plasma Membrane	G-protein coupled receptor
NM_030905.2	OR2J2	olfactory receptor family 2 subfamily J member 2	Plasma	G-protein

			Membrane	coupled receptor
NM_001005470.1	OR4B1	olfactory receptor family 4 subfamily B member 1	Plasma Membrane	G-protein coupled receptor
NM_001005270.2	OR4C12	olfactory receptor family 4 subfamily C member 12	Plasma Membrane	G-protein coupled receptor
NM_001004753.1	OR51F2	olfactory receptor family 51 subfamily F member 2	Plasma Membrane	G-protein coupled receptor
NM_001005238.1	OR51G2	olfactory receptor family 51 subfamily G member 2	Plasma Membrane	G-protein coupled receptor
NM_001004742.1	OR5M3	olfactory receptor family 5 subfamily M member 3	Plasma Membrane	G-protein coupled receptor
NM_001004746.1	OR5T2	olfactory receptor family 5 subfamily T member 2	Plasma Membrane	G-protein coupled receptor
NM_173351.1	OR6B3	olfactory receptor family 6 subfamily B member 3	Plasma Membrane	G-protein coupled receptor
NM_198944.1	OR7C1	olfactory receptor family 7 subfamily C member 1	Plasma Membrane	G-protein coupled receptor
NM_000608.2	ORM2	orosomucoid 2	Extracellular Space	other
NM_030979.2	PABPC3	poly(A) binding protein, cytoplasmic 3	Cytoplasm	other
NM_080832.2	PABPC5	poly(A) binding protein, cytoplasmic 5	Cytoplasm	other
NM_002576.4	PAK1	p21 protein (Cdc42/Rac)-activated kinase 1	Cytoplasm	kinase
NM_152911.2	PAOX	polyamine oxidase (exo-N4-amino)	Cytoplasm	enzyme
NM_178422.4	PAQR7	progestin and adipoQ receptor family member VII	Plasma Membrane	other
NM_006437.3	PARP4	poly(ADP-ribose) polymerase family member 4	Cytoplasm	enzyme
NM_001146106.1	PARP9	poly(ADP-ribose) polymerase family member 9	Nucleus	enzyme
NM_001003828.1	PARVB	parvin beta	Cytoplasm	other
NM_006192.3	PAX1	paired box 1	Nucleus	transcription regulator
NM_001142770.1	PCDH15	protocadherin-related 15	Plasma Membrane	other
NM_031882.2	PCDHAC1	protocadherin alpha subfamily C, 1	Plasma Membrane	other
NM_018936.2	PCDHB2	protocadherin beta 2	Plasma Membrane	other
NM_032373.3	PCGF5	polycomb group ring finger 5	Cytoplasm	other
NM_013232.3	PDCD6	programmed cell death 6	Cytoplasm	other
NM_018945.3	PDE7B	phosphodiesterase 7B	Cytoplasm	enzyme
NM_173806.3	PDZD9	PDZ domain containing 9	Other	other
NM_000442.3	PECAM1	platelet/endothelial cell adhesion molecule 1	Plasma Membrane	other
NM_002627.3	PFKP	phosphofructokinase, platelet	Cytoplasm	kinase
NM_000290.3	PGAM2	phosphoglycerate mutase 2	Cytoplasm	phosphatase
NM_138733.4	PGK2	phosphoglycerate kinase 2	Cytoplasm	kinase
NM_173341.1	PHF7	PHD finger protein 7	Nucleus	other
NM_018425.2	PI4K2A	phosphatidylinositol 4-kinase type 2 alpha	Cytoplasm	kinase
NM_007166.2	PICALM	phosphatidylinositol binding clathrin assembly	Cytoplasm	other

		protein		
NM_178272.1	PILRA	paired immunoglobin-like type 2 receptor alpha	Plasma Membrane	other
NR_002319.2	PIPSL	PIP5K1A and PSMD4-like, pseudogene	Other	other
NM_004571.3	PKNOX1	PBX/knotted 1 homeobox 1	Nucleus	transcription regulator
NM_002657.3	PLAGL2	PLAG1 like zinc finger 2	Nucleus	transcription regulator
NM_020359.1	PLSCR2	phospholipid scramblase 2	Other	other
NM_000534.4	PMS1	PMS1 homolog 1, mismatch repair system component	Nucleus	enzyme
NM_001103149.1	PNMAL1	paraneoplastic Ma antigen family-like 1	Other	other
NM_021173.3	POLD4	polymerase (DNA-directed), delta 4, accessory subunit	Nucleus	enzyme
NM_007215.3	POLG2	polymerase (DNA directed), gamma 2, accessory subunit	Cytoplasm	enzyme
NM_006233.4	POLR2I	polymerase (RNA) II (DNA directed) polypeptide I, 14.5kDa	Nucleus	transcription regulator
NM_001136114.1	POMT1	protein-O-mannosyltransferase 1	Cytoplasm	enzyme
NM_015029.2	POP1	POP1 homolog, ribonuclease P/MRP subunit	Nucleus	enzyme
NM_001145442.1	POTEH (includes others)	POTE ankyrin domain family member M	Other	other
NM_002703.3	PPAT	phosphoribosyl pyrophosphate amidotransferase	Cytoplasm	enzyme
NM_003625.2	PPFIA2	protein tyrosine phosphatase, receptor type, f polypeptide (PTPRF), interacting protein (liprin), alpha 2	Plasma Membrane	phosphatase
NM_021130.3	PPIA	peptidylprolyl isomerase A	Cytoplasm	enzyme
NM_173672.4	PPIL6	peptidylprolyl isomerase like 6	Other	enzyme
NM_177983.1	PPM1G	protein phosphatase, Mg2+/Mn2+ dependent 1G	Nucleus	phosphatase
NM_005167.5	PPM1J	protein phosphatase, Mg2+/Mn2+ dependent 1J	Other	phosphatase
NM_016147.1	PPME1	protein phosphatase methylesterase 1	Other	enzyme
NM_002710.2	PPP1CC	protein phosphatase 1, catalytic subunit, gamma isozyme	Nucleus	phosphatase
NM_006241.4	PPP1R2	protein phosphatase 1 regulatory inhibitor subunit 2	Cytoplasm	phosphatase
NM_001013626.2	PPP1R42	protein phosphatase 1 regulatory subunit 42	Cytoplasm	other
NM_017917.2	PPP2R3C	protein phosphatase 2 regulatory subunit B", gamma	Cytoplasm	other
NM_014678.3	PPP6R2	protein phosphatase 6 regulatory subunit 2	Cytoplasm	other
NM_001012277.1	PRAMEF7/PRAMEF8	PRAME family member 8	Other	other
NM_001136239.1	PRDM6	PR domain containing 6	Nucleus	other
NM_020227.2	PRDM9	PR domain containing 9	Nucleus	enzyme
NM_198859.3	PRICKLE2	prickle planar cell polarity protein 2	Nucleus	other
NM_000947.2	PRIM2	primase, DNA, polypeptide 2 (58kDa)	Nucleus	enzyme
NM_004157.2	PRKAR2A	protein kinase, cAMP-dependent, regulatory subunit type II alpha	Cytoplasm	kinase
NM_145040.2	PRKCDBP	protein kinase C, delta binding protein	Cytoplasm	other
NM_002761.2	PRM1	protamine 1	Nucleus	other
NM_002761.2	PRM1	protamine 1	Nucleus	other
NM_002762.2	PRM2	protamine 2	Nucleus	other
NM_012409.2	PRND	prion protein 2 (dublet)	Plasma Membrane	other
NM_172341.1	PSENEN	presenilin enhancer gamma secretase subunit	Plasma	peptidase

			Membrane	
NM_006742.2	PSKH1	protein serine kinase H1	Nucleus	kinase
NM_002789.4	PSMA4	proteasome subunit alpha 4	Cytoplasm	peptidase
NM_002799.2	PSMB7	proteasome subunit beta 7	Cytoplasm	peptidase
NM_002808.3	PSMD2	proteasome 26S subunit, non-ATPase 2	Cytoplasm	other
NM_020232.3	PSMG2	proteasome (prosome, macropain) assembly chaperone 2	Nucleus	other
NR_023917.1	PTENP1	phosphatase and tensin homolog pseudogene 1 (functional)	Cytoplasm	other
NM_015317.1	PUM2	pumilio RNA binding family member 2	Cytoplasm	other
NM_002854.2	PVALB	parvalbumin	Cytoplasm	other
NM_006505.3	PVR	poliovirus receptor	Plasma Membrane	other
NM_015480.1	PVRL3	poliovirus receptor-related 3	Plasma Membrane	other
NM_015470.2	RAB11FIP5	RAB11 family interacting protein 5 (class I)	Cytoplasm	other
NM_00101797 9.2	RAB28	RAB28, member RAS oncogene family	Plasma Membrane	enzyme
NM_002865.1	RAB2A	RAB2A, member RAS oncogene family	Cytoplasm	enzyme
NM_022456.3	RAB3IP	RAB3A interacting protein	Cytoplasm	other
NM_016577.3	RAB6B	RAB6B, member RAS oncogene family	Cytoplasm	enzyme
NM_013277.3	RACGAP1	Rac GTPase activating protein 1	Cytoplasm	transporter
NM_002877.4	RAD51B	RAD51 paralog B	Nucleus	enzyme
NM_006325.3	RAN	RAN, member RAS oncogene family	Nucleus	enzyme
NM_016492.4	RANGRF	RAN guanine nucleotide release factor	Nucleus	transporter
NM_213589.1	RAPH1	Ras association (RalGDS/AF-6) and pleckstrin homology domains 1	Plasma Membrane	other
NM_004841.3	RASAL2	RAS protein activator like 2	Extracellul ar Space	other
NM_153815.2	RASGRF1	Ras protein specific guanine nucleotide releasing factor 1	Cytoplasm	other
NM_005447.3	RASSF9	Ras association (RalGDS/AF-6) domain family (N-terminal) member 9	Cytoplasm	transporter
NR_015343.1	RBAKDN	RBAK downstream neighbor (non-protein coding)	Other	other
NM_203390.2	RBM12B	RNA binding motif protein 12B	Other	other
NM_016196.3	RBM19	RNA binding motif protein 19	Nucleus	other
NM_018047.2	RBM22	RNA binding motif protein 22	Nucleus	transporter
NM_025232.2	REEP4	receptor accessory protein 4	Cytoplasm	other
NM_138393.1	REEP6	receptor accessory protein 6	Plasma Membrane	other
NM_002913.3	RFC1	replication factor C subunit 1	Nucleus	transcription regulator
NM_003721.2	RFXANK	regulatory factor X associated ankyrin containing protein	Nucleus	transcription regulator
NM_183353.2	RLIM	ring finger protein, LIM domain interacting	Nucleus	enzyme
NM_006911.2	RLN1	relaxin 1	Extracellul ar Space	other
NM_145250.3	RNASE11	ribonuclease, RNase A family, 11 (non-active)	Extracellul ar Space	other
NM_016422.3	RNF141	ring finger protein 141	Other	enzyme
NM_016494.3	RNF181	ring finger protein 181	Other	enzyme
NM_00114668 4.2	RNF222	ring finger protein 222	Other	other
NM_017578.2	ROPN1	rhophilin associated tail protein 1	Cytoplasm	other
NM_080746.2	RPL10L	ribosomal protein L10 like	Nucleus	other
NM_000995.3	RPL34	ribosomal protein L34	Cytoplasm	other

NM_183005.3	RPP38	ribonuclease P/MRP 38kDa subunit	Nucleus	enzyme
NM_152732.3	RSPH9	radial spoke head 9 homolog (Chlamydomonas)	Other	other
NM_001172509.1	SATB2	SATB homeobox 2	Nucleus	transcription regulator
NM_001160160.1	SCN5A	sodium channel, voltage gated, type V alpha subunit	Plasma Membrane	ion channel
NM_001130413.1	SCNN1D	sodium channel, non voltage gated 1 delta subunit	Plasma Membrane	ion channel
NM_003000.2	SDHB	succinate dehydrogenase complex subunit B, iron sulfur (Ip)	Cytoplasm	enzyme
NM_006378.3	SEMA4D	semaphorin 4D	Plasma Membrane	transmembrane receptor
NM_003008.2	SEMG2	semenogelin II	Extracellular Space	other
NM_021627.2	SENP2	SUMO1/sentrin/SMT3 specific peptidase 2	Nucleus	peptidase
NM_020654.3	SENP7	SUMO1/sentrin specific peptidase 7	Nucleus	peptidase
NM_144605.3	SEPT12	septin 12	Cytoplasm	other
NR_024271.1	SEPT7P2	septin 7 pseudogene 2	Other	other
NM_032861.3	SERAC1	serine active site containing 1	Extracellular Space	other
NM_004568.4	SERPINB6	serpin peptidase inhibitor, clade B (ovalbumin), member 6	Cytoplasm	other
NM_144665.2	SESN3	sestrin 3	Extracellular Space	other
NM_199344.2	SFT2D2	SFT2 domain containing 2	Cytoplasm	other
NM_032740.3	SFT2D3	SFT2 domain containing 3	Other	other
NM_198843.2	SFTPB	surfactant protein B	Extracellular Space	other
NM_001099289.1	SH3RF3	SH3 domain containing ring finger 3	Other	other
NM_023068.3	SIGLEC1	sialic acid binding Ig-like lectin 1, sialoadhesin	Plasma Membrane	other
NM_033130.4	SIGLEC10	sialic acid binding Ig-like lectin 10	Plasma Membrane	other
NM_015073.1	SIPA1L3	signal-induced proliferation-associated 1 like 3	Extracellular Space	other
NM_004694.4	SLC16A6	solute carrier family 16 member 6	Plasma Membrane	transporter
NM_004731.3	SLC16A7	solute carrier family 16 (monocarboxylate transporter), member 7	Plasma Membrane	transporter
NM_005071.1	SLC1A6	solute carrier family 1 (high affinity aspartate/glutamate transporter), member 6	Plasma Membrane	transporter
NM_002555.5	SLC22A18	solute carrier family 22 member 18	Plasma Membrane	transporter
NM_012140.3	SLC25A10	solute carrier family 25 (mitochondrial carrier; dicarboxylate transporter), member 10	Cytoplasm	transporter
NM_021734.4	SLC25A19	solute carrier family 25 (mitochondrial thiamine pyrophosphate carrier), member 19	Cytoplasm	transporter
NM_031291.2	SLC25A31	solute carrier family 25 (mitochondrial carrier; adenine nucleotide translocator), member 31	Cytoplasm	transporter
NM_138773.1	SLC25A46	solute carrier family 25 member 46	Cytoplasm	other
NM_145282.4	SLC25A48	solute carrier family 25 member 48	Other	other
NM_005094.3	SLC27A4	solute carrier family 27 (fatty acid transporter), member 4	Plasma Membrane	transporter
NM_005094.3	SLC27A4	solute carrier family 27 (fatty acid transporter), member 4	Plasma Membrane	transporter
NM_003039.2	SLC2A5	solute carrier family 2 (facilitated glucose/fructose transporter), member 5	Plasma Membrane	transporter
NM_014580.3	SLC2A8	solute carrier family 2 (facilitated glucose	Plasma	transporter

		transporter), member 8	Membrane	
NM_001177998.1	SLC34A2	solute carrier family 34 (type II sodium/phosphate cotransporter), member 2	Plasma Membrane	transporter
NM_138570.2	SLC38A10	solute carrier family 38 member 10	Cytoplasm	other
NM_144564.4	SLC39A3	solute carrier family 39 (zinc transporter), member 3	Plasma Membrane	transporter
NM_003615.3	SLC4A7	solute carrier family 4, sodium bicarbonate cotransporter, member 7	Plasma Membrane	transporter
NM_144990.3	SLFNL1	schlafen like 1	Other	other
NM_001127217.2	SMAD9	SMAD family member 9	Nucleus	transcription regulator
NM_017575.4	SMG6	SMG6 nonsense mediated mRNA decay factor	Nucleus	enzyme
NM_022739.3	SMURF2	SMAD specific E3 ubiquitin protein ligase 2	Cytoplasm	enzyme
NM_004782.3	SNAP29	synaptosome associated protein 29kDa	Cytoplasm	transporter
NR_029472.1	SNRPC	small nuclear ribonucleoprotein polypeptide C	Nucleus	other
NM_182854.2	SNX20	sorting nexin 20	Cytoplasm	other
NM_080627.2	SOGA1	suppressor of glucose, autophagy associated 1	Extracellular Space	other
NM_017425.3	SPA17	sperm autoantigenic protein 17	Plasma Membrane	other
NM_006461.3	SPAG5	sperm associated antigen 5	Nucleus	peptidase
NM_001130528.2	SPAG9	sperm associated antigen 9	Cytoplasm	other
NM_138796.2	SPATA17	spermatogenesis associated 17	Other	other
NM_001170696.1	SPATA22	spermatogenesis associated 22	Other	other
NM_001170696.1	SPATA22	spermatogenesis associated 22	Other	other
NM_194296.1	SPATA24	spermatogenesis associated 24	Nucleus	other
NM_173499.3	SPATA8	spermatogenesis associated 8	Other	other
NM_001100422.1	SPATS2L	spermatogenesis associated, serine rich 2 like	Nucleus	other
NM_014752.2	SPCS2	signal peptidase complex subunit 2	Cytoplasm	other
NM_024867.3	SPEF2	sperm flagellar 2	Cytoplasm	other
NM_145658.3	SPESP1	sperm equatorial segment protein 1	Cytoplasm	other
NM_006542.3	SPHAR	S-phase response (cyclin related)	Other	other
NM_021114.2	SPINK2	serine peptidase inhibitor, Kazal type 2 (acrosin-trypsin inhibitor)	Extracellular Space	other
NM_021102.3	SPINT2	serine peptidase inhibitor, Kunitz type, 2	Extracellular Space	other
NM_020148.2	SPIRE1	spire-type actin nucleation factor 1	Cytoplasm	other
NM_001042522.1	SPRED3	sprouty-related, EVH1 domain containing 3	Extracellular Space	cytokine
NM_198291.1	SRC	SRC proto-oncogene, non-receptor tyrosine kinase	Cytoplasm	kinase
NM_015908.5	SRRT	serrate, RNA effector molecule	Nucleus	other
NM_006275.5	SRSF6	serine/arginine-rich splicing factor 6	Nucleus	other
NM_015931.1	SSUH2	ssu-2 homolog (C. elegans)	Cytoplasm	other
NM_012447.2	STAG3	stromal antigen 3	Nucleus	other
NM_139171.1	STARD6	StAR related lipid transfer domain containing 6	Other	transporter
NM_004226.3	STK17B	serine/threonine kinase 17b	Nucleus	kinase
NM_032017.1	STK40	serine/threonine kinase 40	Cytoplasm	kinase
NM_001165969.1	STRADA	STE20-related kinase adaptor alpha	Nucleus	kinase
NM_178862.1	STT3B	STT3B, subunit of the oligosaccharyltransferase complex (catalytic)	Cytoplasm	enzyme
NM_016086.2	STYXL1	serine/threonine/tyrosine interacting-like 1	Cytoplasm	phosphatase

NM_003849.3	SUCLG1	succinate-CoA ligase, alpha subunit	Cytoplasm	enzyme
NM_003169.3	SUPT5H	SPT5 homolog, DSIF elongation factor subunit	Nucleus	transcription regulator
NM_001105518.1	SWT1	SWT1, RNA endoribonuclease homolog	Extracellular Space	other
NM_001123225.1	SYCE3	synaptonemal complex central element protein 3	Nucleus	other
NM_153694.4	SYCP3	synaptonemal complex protein 3	Nucleus	other
NM_001033080.1	TAAR2	trace amine associated receptor 2	Plasma Membrane	G-protein coupled receptor
NM_006284.2	TAF10	TATA-box binding protein associated factor 10	Nucleus	transcription regulator
NM_005640.1	TAF4B	TATA-box binding protein associated factor 4b	Nucleus	transcription regulator
NM_016151.2	TAOK2	TAO kinase 2	Cytoplasm	kinase
NM_144628.2	TBC1D20	TBC1 domain family member 20	Nucleus	other
NM_153356.1	TBC1D21	TBC1 domain family member 21	Cytoplasm	other
NM_001029839.2	TCAIM	T-cell activation inhibitor, mitochondrial	Cytoplasm	other
NR_002947.1	TCAM1P	testicular cell adhesion molecule 1, pseudogene	Other	other
NM_003202.3	TCF7	transcription factor 7 (T-cell specific, HMG-box)	Nucleus	transcription regulator
NM_006602.2	TCFL5	transcription factor-like 5 (basic helix-loop-helix)	Nucleus	transcription regulator
NM_018679.4	TCP11	t-complex 11, testis-specific	Other	other
NM_018679.4	TCP11	t-complex 11, testis-specific	Other	other
NM_138779.3	TEX30	testis expressed 30	Other	other
NM_001039496.1	TEX40	testis expressed 40	Cytoplasm	other
NM_001178138.1	TFDP2	transcription factor Dp-2 (E2F dimerization partner 2)	Nucleus	transcription regulator
NM_024672.4	THAP9	THAP domain containing 9	Other	enzyme
NM_017872.3	THG1L	tRNA-histidine guanylyltransferase 1-like	Cytoplasm	enzyme
NM_145715.2	TIGD2	tigger transposable element derived 2	Other	other
NM_007005.3	TLE4	transducin like enhancer of split 4	Nucleus	transcription regulator
NM_016056.2	TMBIM4	transmembrane BAX inhibitor motif containing 4	Nucleus	other
NM_080751.2	TMC2	transmembrane channel like 2	Plasma Membrane	ion channel
NM_144686.2	TMC4	transmembrane channel like 4	Cytoplasm	other
NR_030761.1	TMED5	transmembrane p24 trafficking protein 5	Cytoplasm	other
NM_178031.2	TMEM132A	transmembrane protein 132A	Cytoplasm	other
NM_194280.3	TMEM219	transmembrane protein 219	Other	other
NM_152388.2	TMEM237	transmembrane protein 237	Other	other
NR_027428.1	TMEM254-AS1	TMEM254 antisense RNA 1	Other	other
NM_182541.2	TMEM31	transmembrane protein 31	Other	other
NM_018022.2	TMEM51	transmembrane protein 51	Other	other
NM_182559.2	TMPRSS12	transmembrane (C-terminal) protease, serine 12	Other	peptidase
NM_001080495.2	TNRC18	trinucleotide repeat containing 18	Nucleus	other
NM_001162501.1	TNRC6B	trinucleotide repeat containing 6B	Other	other
NM_015476.2	TPGS2	tubulin polyglutamylase complex subunit 2	Nucleus	other
NM_173846.4	TPPP2	tubulin polymerization-promoting protein family member 2	Nucleus	other
NM_003292.2	TPR	translocated promoter region, nuclear basket	Nucleus	transporter

		protein		
NM_199259.2	TPTE	transmembrane phosphatase with tensin homology	Plasma Membrane	phosphatase
NM_014939.3	TRAPPC8	trafficking protein particle complex 8	Cytoplasm	transporter
NM_178174.2	TREML1	triggering receptor expressed on myeloid cells like 1	Plasma Membrane	other
NM_018700.3	TRIM36	tripartite motif containing 36	Cytoplasm	other
NM_033092.2	TRIM5	tripartite motif containing 5	Cytoplasm	enzyme
NM_001171796.1	TRIQK	triple QxxK/R motif containing	Other	other
NM_018646.2	TRPV6	transient receptor potential cation channel, subfamily V, member 6	Plasma Membrane	ion channel
NM_144627.2	TSACC	TSSK6 activating co-chaperone	Cytoplasm	other
NM_006292.2	TSG101	tumor susceptibility 101	Cytoplasm	transcription regulator
NM_018430.2	TSNAXIP1	translin-associated factor X interacting protein 1	Cytoplasm	other
NM_003270.2	TSPAN6	tetraspanin 6	Plasma Membrane	other
NM_003309.3	TSPYL1	TSPY-like 1	Nucleus	other
NM_052841.3	TSSK3	testis-specific serine kinase 3	Other	kinase
NM_174944.3	TSSK4	testis-specific serine kinase 4	Cytoplasm	kinase
NM_001105513.2	TTC21A	tetratricopeptide repeat domain 21A	Extracellular Space	other
NM_153712.4	TTL	tubulin tyrosine ligase	Cytoplasm	enzyme
NM_173623.3	TTLL6	tubulin tyrosine ligase like 6	Other	enzyme
NM_006088.5	TUBB4B	tubulin beta 4B class IVb	Cytoplasm	other
NM_052903.4	TUBGCP5	tubulin gamma complex associated protein 5	Cytoplasm	other
NM_003321.4	TUFM	Tu translation elongation factor, mitochondrial	Cytoplasm	translation regulator
NR_002323.1	TUG1	taurine up-regulated 1 (non-protein coding)	Other	other
NM_018955.2	UBB	ubiquitin B	Cytoplasm	enzyme
NM_005339.4	UBE2K	ubiquitin conjugating enzyme E2K	Cytoplasm	transcription regulator
NM_022066.3	UBE2O	ubiquitin conjugating enzyme E2O	Nucleus	enzyme
NM_198920.1	UBE3D	ubiquitin protein ligase E3D	Other	other
NM_021833.4	UCP1	uncoupling protein 1 (mitochondrial, proton carrier)	Cytoplasm	transporter
NR_028085.1	UFSP2	UFM1-specific peptidase 2	Other	enzyme
NM_001105677.1	UGT2A2	UDP glucuronosyltransferase 2 family, polypeptide A2	Other	other
NM_001080461.1	UNCX	UNC homeobox	Other	transcription regulator
NM_007124.2	UTRN	utrophin	Plasma Membrane	transmembrane receptor
NM_001017980.3	VMA21	VMA21 vacuolar H+-ATPase homolog (S. cerevisiae)	Cytoplasm	other
NM_181661.2	VPS13B	vacuolar protein sorting 13 homolog B (yeast)	Nucleus	transporter
NM_015289.2	VPS39	VPS39 HOPS complex subunit	Cytoplasm	transporter
NM_024626.2	VTCN1	V-set domain containing T cell activation inhibitor 1	Plasma Membrane	other
NM_001024935.1	WASF1	WAS protein family member 1	Nucleus	other
NM_001006657.1	WDR35	WD repeat domain 35	Cytoplasm	other
NM_032118.2	WDR54	WD repeat domain 54	Other	other
NM_133264.4	WIPF2	WAS/WASL interacting protein family member 2	Cytoplasm	other

NM_052898.1	XKR4	X-linked Kx blood group related 4	Other	other
NM_004736.3	XPR1	xenotropic and polytropic retrovirus receptor 1	Plasma Membrane	G-protein coupled receptor
NM_001006114.1	YBEY	ybeY metallopeptidase (putative)	Cytoplasm	other
NM_004559.3	YBX1	Y-box binding protein 1	Nucleus	transcription regulator
NM_024029.3	YIPF2	Yip1 domain family member 2	Cytoplasm	other
NM_182592.2	YIPF7	Yip1 domain family member 7	Other	other
NM_198537.2	YJEFN3	YjeF N-terminal domain containing 3	Other	other
NM_017798.3	YTHDF1	YTH N(6)-methyladenosine RNA binding protein 1	Other	other
NM_001105539.1	ZBTB10	zinc finger and BTB domain containing 10	Nucleus	other
NM_001164342.1	ZBTB20	zinc finger and BTB domain containing 20	Nucleus	transcription regulator
NM_032792.2	ZBTB45	zinc finger and BTB domain containing 45	Nucleus	other
NM_207662.3	ZC3H14	zinc finger CCCH-type containing 14	Nucleus	other
NM_001128324.1	ZFAND4	zinc finger, AN1-type domain 4	Other	other
NM_053023.3	ZFP91	ZFP91 zinc finger protein	Nucleus	transcription regulator
XM_001724926.2	ZMYM6NB	ZMYM6 neighbor	Other	other
NM_021047.2	ZNF253	zinc finger protein 253	Nucleus	transcription regulator
NM_012482.3	ZNF281	zinc finger protein 281	Nucleus	transcription regulator
NM_170686.2	ZNF398	zinc finger protein 398	Nucleus	transcription regulator
NM_001001415.2	ZNF429	zinc finger protein 429	Nucleus	other
NM_025189.3	ZNF430	zinc finger protein 430	Nucleus	other
NM_001101419.1	ZNF541	zinc finger protein 541	Nucleus	other
NM_145271.3	ZNF688	zinc finger protein 688	Other	other
NM_206894.2	ZNF790	zinc finger protein 790	Other	other
NM_004773.2	ZNHIT3	zinc finger, HIT-type containing 3	Nucleus	transcription regulator
NM_147128.3	ZNRF2	zinc and ring finger 2, E3 ubiquitin protein ligase	Other	enzyme
NM_003904.3	ZPR1	ZPR1 zinc finger	Nucleus	other
NM_152677.2	ZSCAN4	zinc finger and SCAN domain containing 4	Nucleus	other
NM_006299.3	ZSCAN9	zinc finger and SCAN domain containing 9	Nucleus	transcription regulator
XM_001133704.1		unknown	unknown	unknown
XM_001714193.1		unknown	unknown	unknown
XM_001714387.2		unknown	unknown	unknown
XM_001716063.1		unknown	unknown	unknown
XM_001719118.1		unknown	unknown	unknown
XM_001725120.2		unknown	unknown	unknown
XM_001725120.2		unknown	unknown	unknown

XM_002344166.1		unknown	unknown	unknown
XM_002347401.1		unknown	unknown	unknown
XM_002347847.1		unknown	unknown	unknown
XM_934920.4		unknown	unknown	unknown
XR_038906.2		unknown	unknown	unknown
XR_039130.2		unknown	unknown	unknown
XR_040674.1		unknown	unknown	unknown
XR_042117.1		unknown	unknown	unknown
XR_078317.1		unknown	unknown	unknown
XR_078370.1		unknown	unknown	unknown
XR_078671.1		unknown	unknown	unknown
XR_079298.1		unknown	unknown	unknown

Supplementary Table S2. Up-regulated Genes in Cluster B

**ID**	**Symbol**	**Entrez Gene Name**	**Location**	**Type(s)**
NM_015429.3	ABI3BP	ABI family member 3 binding protein	Extracellular Space	other
NM_014384.2	ACAD8	acyl-CoA dehydrogenase family member 8	Cytoplasm	enzyme
NM_014716.3	ACAP1	ArfGAP with coiled-coil, ankyrin repeat and PH domains 1	Plasma Membrane	other
NM_025149.4	ACSF2	acyl-CoA synthetase family member 2	Cytoplasm	enzyme
NM_177989.2	ACTL6A	actin like 6A	Nucleus	other
NM_004302.3	ACVR1B	activin A receptor type IB	Plasma Membrane	kinase
NM_001169122.1	AFF2	AF4/FMR2 family member 2	Nucleus	other
NM_000476.2	AK1	adenylate kinase 1	Cytoplasm	kinase
NM_000690.2	ALDH2	aldehyde dehydrogenase 2 family (mitochondrial)	Cytoplasm	enzyme
NM_001127617.1	ALDOA	aldolase, fructose-bisphosphate A	Cytoplasm	enzyme
NR_024400.1	ANAPC13	anaphase promoting complex subunit 13	Nucleus	other
NM_052855.3	ANKRD40	ankyrin repeat domain 40	Other	other
NM_001154.3	ANXA5	annexin A5	Plasma Membrane	transporter
NM_001159.3	AOX1	aldehyde oxidase 1	Cytoplasm	enzyme
NM_144772.2	APOA1BP	apolipoprotein A-I binding protein	Extracellular Space	enzyme
NM_175073.1	APTX	aprataxin	Nucleus	phosphatase
NM_006407.3	ARL6IP5	ADP ribosylation factor like GTPase 6 interacting protein 5	Cytoplasm	other
NM_001030287.2	ATF3	activating transcription factor 3	Nucleus	transcription regulator
NM_005174.2	ATP5C1	ATP synthase, H+ transporting, mitochondrial F1 complex, gamma polypeptide 1	Cytoplasm	transporter
NM_004048.2	B2M	beta-2-microglobulin	Plasma Membrane	transmembrane receptor
NM_001024372.1	BAALC	brain and acute leukemia, cytoplasmic	Cytoplasm	other
NM_000633.2	BCL2	B-cell CLL/lymphoma 2	Cytoplasm	transporter
NM_007371.3	BRD3	bromodomain containing 3	Nucleus	kinase

NM_198591.1	BSG	basigin (Ok blood group)	Plasma Membrane	transporter
NM_181443.1	BTBD3	BTB (POZ) domain containing 3	Other	other
NM_006763.2	BTG2	BTG family member 2	Nucleus	transcription regulator
NM_001145194.1	C18orf42	chromosome 18 open reading frame 42	Other	other
NM_001105519.1	C2orf70	chromosome 2 open reading frame 70	Nucleus	other
NM_032307.3	C9orf64	chromosome 9 open reading frame 64	Other	other
NM_001740.4	CALB2	calbindin 2	Cytoplasm	other
NM_006136.2	CAPZA2	capping protein (actin filament) muscle Z-line, alpha 2	Cytoplasm	other
NM_014550.3	CARD10	caspase recruitment domain family member 10	Cytoplasm	other
NM_138423.2	CASC4	cancer susceptibility candidate 4	Cytoplasm	other
NM_001172895.1	CAV1	caveolin 1	Plasma Membrane	transmembrane receptor
NM_001031737.2	CCDC78	coiled-coil domain containing 78	Cytoplasm	other
NM_001136017.2	CCND3	cyclin D3	Nucleus	other
NM_012073.3	CCT5	chaperonin containing TCP1 subunit 5	Cytoplasm	other
NM_006725.3	CD6	CD6 molecule	Plasma Membrane	transmembrane receptor
NM_001040034.1	CD63	CD63 molecule	Plasma Membrane	other
NM_002414.3	CD99	CD99 molecule	Plasma Membrane	other
NM_001791.3	CDC42	cell division cycle 42	Cytoplasm	enzyme
NM_001257.3	CDH13	cadherin 13	Plasma Membrane	other
NM_005195.3	CEBPD	CCAAT/enhancer binding protein delta	Nucleus	transcription regulator
NM_004344.1	CETN2	centrin 2	Nucleus	enzyme
NM_001710.5	CFB	complement factor B	Extracellular Space	peptidase
NM_001928.2	CFD	complement factor D (adipsin)	Extracellular Space	peptidase
NM_001928.2	CFD	complement factor D (adipsin)	Extracellular Space	peptidase
NM_000204.3	CFI	complement factor I	Extracellular Space	peptidase
NM_001005753.1	CHMP3	charged multivesicular body protein 3	Cytoplasm	other
NM_015455.3	CNOT6	CCR4-NOT transcription complex subunit 6	Nucleus	enzyme
NM_015719.3	COL5A3	collagen, type V, alpha 3	Extracellular Space	other
NM_004074.2	COX8A	cytochrome c oxidase subunit VIIIA (ubiquitous)	Cytoplasm	enzyme
NM_003476.3	CSRP3	cysteine and glycine rich protein 3	Nucleus	other
NM_000100.2	CSTB	cystatin B	Cytoplasm	peptidase
NM_021198.1	CTDSP1	CTD small phosphatase 1	Nucleus	phosphatase
NM_148923.2	CYB5A	cytochrome b5 type A (microsomal)	Cytoplasm	enzyme
NM_134268.3	CYGB	cytoglobin	Cytoplasm	transporter
NM_000781.2	CYP11A1	cytochrome P450 family 11 subfamily A member 1	Cytoplasm	enzyme
NM_000102.3	CYP17A1	cytochrome P450 family 17 subfamily A member 1	Cytoplasm	enzyme
NM_001348.1	DAPK3	death-associated protein kinase 3	Cytoplasm	kinase
NM_001923.3	DDB1	damage-specific DNA binding protein 1	Nucleus	other
NM_018706.5	DHTKD1	dehydrogenase E1 and transketolase domain containing 1	Cytoplasm	enzyme

NM_021931.2	DHX35	DEAH-box helicase 35	Other	enzyme
NM_003836.5	DLK1	delta-like 1 homolog (Drosophila)	Extracellular Space	other
NM_015190.3	DNAJC9	DnaJ heat shock protein family (Hsp40) member C9	Nucleus	other
NM_139159.4	DPP9	dipeptidyl-peptidase 9	Cytoplasm	peptidase
NM_001388.3	DRG2	developmentally regulated GTP binding protein 2	Cytoplasm	other
NM_006870.3	DSTN	destrin (actin depolymerizing factor)	Cytoplasm	other
NM_004417.3	DUSP1	dual specificity phosphatase 1	Nucleus	phosphatase
NM_030640.2	DUSP16	dual specificity phosphatase 16	Nucleus	phosphatase
NM_004418.3	DUSP2	dual specificity phosphatase 2	Nucleus	phosphatase
NM_014183.2	DYNLRB1	dynein, light chain, roadblock-type 1	Cytoplasm	other
NM_024007.3	EBF1	early B-cell factor 1	Nucleus	transcription regulator
NM_016938.3	EFEMP2	EGF containing fibulin-like extracellular matrix protein 2	Extracellular Space	other
NM_022051.2	EGLN1	egl-9 family hypoxia-inducible factor 1	Cytoplasm	enzyme
NM_001964.2	EGR1	early growth response 1	Nucleus	transcription regulator
NM_004681.2	EIF1AY	eukaryotic translation initiation factor 1A, Y-linked	Other	translation regulator
NM_032025.3	EIF2A	eukaryotic translation initiation factor 2A	Cytoplasm	translation regulator
NM_001247.2	ENTPD6	ectonucleoside triphosphate diphosphohydrolase 6 (putative)	Cytoplasm	enzyme
NM_006817.3	ERP29	endoplasmic reticulum protein 29	Cytoplasm	transporter
NM_004730.2	ETF1	eukaryotic translation termination factor 1	Cytoplasm	translation regulator
NM_001162422.1	ETS1	v-ets avian erythroblastosis virus E26 oncogene homolog 1	Nucleus	transcription regulator
NM_005239.4	ETS2	v-ets avian erythroblastosis virus E26 oncogene homolog 2	Nucleus	transcription regulator
NM_007177.2	FAM107A	family with sequence similarity 107 member A	Nucleus	other
NM_014612.3	FAM120A	family with sequence similarity 120A	Cytoplasm	other
NM_176782.2	FAM151A	family with sequence similarity 151 member A	Extracellular Space	other
NM_004111.4	FEN1	flap structure-specific endonuclease 1	Nucleus	enzyme
NM_006832.2	FERMT2	fermitin family member 2	Cytoplasm	other
NM_207422.2	FLJ44635	TPT1-like protein	Cytoplasm	other
NM_013280.4	FLRT1	fibronectin leucine rich transmembrane protein 1	Plasma Membrane	other
NM_212475.1	FN1	fibronectin 1	Extracellular Space	enzyme
NM_001114171.1	FOSB	FBJ murine osteosarcoma viral oncogene homolog B	Nucleus	transcription regulator
NM_001453.2	FOXC1	forkhead box C1	Nucleus	transcription regulator
NR_002201.1	FTH1P3	ferritin, heavy polypeptide 1 pseudogene 3	Other	other
NM_000146.3	FTL	ferritin, light polypeptide	Cytoplasm	enzyme
NR_028388.2	FUS	FUS RNA binding protein	Nucleus	transcription regulator
NM_006581.3	FUT9	fucosyltransferase 9 (alpha (1,3) fucosyltransferase)	Cytoplasm	enzyme
NM_054110.4	GALNT15	polypeptide N-acetylgalactosaminyltransferase 15	Cytoplasm	enzyme
NR_027399.1	GFOD2	glucose-fructose oxidoreductase domain containing 2	Extracellular Space	enzyme
NM_000165.3	GJA1	gap junction protein alpha 1	Plasma Membrane	transporter
NM_016194.3	GNB5	guanine nucleotide binding protein (G protein), beta 5	Plasma Membrane	enzyme

NM_002076.3	GNS	glucosamine (N-acetyl)-6-sulfatase	Cytoplasm	enzyme
NM_016548.3	GOLM1	golgi membrane protein 1	Cytoplasm	other
NM_022130.3	GOLPH3	golgi phosphoprotein 3 (coat-protein)	Cytoplasm	other
NM_005814.1	GPA33	glycoprotein A33	Plasma Membrane	other
NM_000581.2	GPX1	glutathione peroxidase 1	Cytoplasm	enzyme
NM_005315.1	GSC2	goosecoid homeobox 2	Nucleus	transcription regulator
NM_001127662.1	GSN	gelsolin	Extracellular Space	other
NM_000846.4	GSTA2	glutathione S-transferase alpha 2	Cytoplasm	enzyme
NM_002101.3	GYPC	glycophorin C (Gerbich blood group)	Plasma Membrane	other
NR_002196.1	H19	H19, imprinted maternally expressed transcript (non-protein coding)	Cytoplasm	other
NM_001010989.1	HERPUD1	homocysteine-inducible, endoplasmic reticulum stress-inducible, ubiquitin-like domain member 1	Cytoplasm	other
NM_144608.1	HEXIM2	hexamethylene bis-acetamide inducible 2	Nucleus	transcription regulator
NM_002116.6	HLA-A	major histocompatibility complex, class I, A	Plasma Membrane	other
NM_019111.3	HLA-DRA	major histocompatibility complex, class II, DR alpha	Plasma Membrane	transmembrane receptor
NM_001098272.1	HMGCS1	3-hydroxy-3-methylglutaryl-CoA synthase 1	Cytoplasm	enzyme
NM_001123366.1	HMSD	histocompatibility (minor) serpin domain containing	Other	other
NR_027297.1	HOMER3	homer scaffolding protein 3	Plasma Membrane	other
NM_006042.1	HS3ST3A1	heparan sulfate-glucosamine 3-sulfotransferase 3A1	Cytoplasm	enzyme
NM_003725.2	HSD17B6	hydroxysteroid (17-beta) dehydrogenase 6	Other	enzyme
NM_002156.4	HSPD1	heat shock protein family D (Hsp60) member 1	Cytoplasm	enzyme
NM_003897.3	IER3	immediate early response 3	Cytoplasm	other
NM_206949.2	IFI27L1	interferon, alpha-inducible protein 27-like 1	Other	other
NM_032036.2	IFI27L2	interferon, alpha-inducible protein 27-like 2	Other	other
NM_001548.3	IFIT1	interferon induced protein with tetratricopeptide repeats 1	Cytoplasm	other
NM_000877.2	IL1R1	interleukin 1 receptor, type I	Plasma Membrane	transmembrane receptor
NM_002192.2	INHBA	inhibin beta A	Extracellular Space	growth factor
NM_017759.4	INO80D	INO80 complex subunit D	Other	other
NR_003512.1	INS-IGF2	INS-IGF2 readthrough	Other	other
NM_198336.2	INSIG1	insulin induced gene 1	Cytoplasm	other
NM_001034841.3	ITPRIPL2	inositol 1,4,5-trisphosphate receptor interacting protein-like 2	Other	other
NM_002228.3	JUN	jun proto-oncogene	Nucleus	transcription regulator
NM_005354.4	JUND	jun D proto-oncogene	Nucleus	transcription regulator
NM_138444.3	KCTD12	potassium channel tetramerization domain containing 12	Plasma Membrane	ion channel
NM_020702.3	KIAA1161	KIAA1161	Nucleus	other
NM_016270.2	KLF2	Kruppel-like factor 2	Nucleus	transcription regulator
NM_006148.2	LASP1	LIM and SH3 protein 1	Cytoplasm	transporter
NM_001174097.1	LDHB	lactate dehydrogenase B	Cytoplasm	enzyme

NM_000527.3	LDLR	low density lipoprotein receptor	Plasma Membrane	transporter
NM_024316.1	LENG1	leukocyte receptor cluster (LRC) member 1	Other	other
NR_027129.1	LINC01547	long intergenic non-protein coding RNA 1547	Cytoplasm	other
NR_028386.1	LOC375196	uncharacterized LOC375196	Other	other
NM_015578.2	LSM14A	LSM14A mRNA processing body assembly factor	Cytoplasm	other
NM_001033667.1	LY9	lymphocyte antigen 9	Plasma Membrane	other
NM_181705.2	LYRM7	LYR motif containing 7	Cytoplasm	other
NM_153267.4	MAMDC2	MAM domain containing 2	Extracellular Space	other
NM_001177466.1	MAMLD1	mastermind like domain containing 1	Other	other
NM_005909.3	MAP1B	microtubule associated protein 1B	Cytoplasm	other
NM_025052.3	MAP3K19	mitogen-activated protein kinase kinase kinase 19	Other	kinase
NM_002382.3	MAX	MYC associated factor X	Nucleus	transcription regulator
NM_201542.3	MED8	mediator complex subunit 8	Nucleus	other
NM_014033.3	METTL7A	methyltransferase like 7A	Cytoplasm	other
NR_024275.1	MGAT5	mannosyl (alpha-1,6-)-glycoprotein beta-1,6-N-acetyl-glucosaminyltransferase	Cytoplasm	enzyme
NR_027906.1	MLLT4-AS1	MLLT4 antisense RNA 1 (head to head)	Other	other
NR_002946.1	MMP23A	matrix metallopeptidase 23A (pseudogene)	Extracellular Space	other
NM_145330.2	MRPL33	mitochondrial ribosomal protein L33	Cytoplasm	other
NM_006745.3	MSMO1	methylsterol monooxygenase 1	Cytoplasm	enzyme
NM_016332.2	MSRB1	methionine sulfoxide reductase B1	Other	other
NM_004686.4	MTMR7	myotubularin related protein 7	Cytoplasm	phosphatase
NM_001008528.1	MXRA7	matrix-remodelling associated 7	Other	other
NM_021019.3	MYL6	myosin light chain 6	Cytoplasm	other
NM_021019.3	MYL6	myosin light chain 6	Cytoplasm	other
NM_001161819.1	MYO1B	myosin IB	Cytoplasm	other
NM_181351.3	NCAM1	neural cell adhesion molecule 1	Plasma Membrane	other
NM_002486.4	NCBP1	nuclear cap binding protein subunit 1	Nucleus	other
NM_181782.3	NCOA7	nuclear receptor coactivator 7	Nucleus	transcription regulator
NM_006312.3	NCOR2	nuclear receptor corepressor 2	Nucleus	transcription regulator
NM_201535.1	NDRG2	NDRG family member 2	Cytoplasm	other
NM_031232.3	NECAB3	N-terminal EF-hand calcium binding protein 3	Cytoplasm	other
NM_003204.2	NFE2L1	nuclear factor, erythroid 2 like 1	Nucleus	transcription regulator
NM_001134673.3	NFIA	nuclear factor I/A	Nucleus	transcription regulator
NM_014380.1	NGFRAP1	nerve growth factor receptor (TNFRSF16) associated protein 1	Cytoplasm	other
NM_004808.2	NMT2	N-myristoyltransferase 2	Cytoplasm	enzyme
NM_003995.3	NPR2	natriuretic peptide receptor 2	Plasma Membrane	G-protein coupled receptor
NM_001144772.1	NSMAF	neutral sphingomyelinase activation associated factor	Cytoplasm	other

NM_001081491.1	NXF1	nuclear RNA export factor 1	Nucleus	transporter
NM_033014.2	OGN	osteoglycin	Extracellular Space	growth factor
NM_181672.2	OGT	O-linked N-acetylglucosamine (GlcNAc) transferase	Cytoplasm	enzyme
NM_001005471.1	OR2T6	olfactory receptor family 2 subfamily T member 6	Plasma Membrane	G-protein coupled receptor
NM_001004699.1	OR2Z1	olfactory receptor family 2 subfamily Z member 1	Plasma Membrane	G-protein coupled receptor
NM_002553.2	ORC5	origin recognition complex subunit 5	Nucleus	other
NM_002556.2	OSBP	oxysterol binding protein	Cytoplasm	transporter
NM_001136157.1	OTUD5	OTU deubiquitinase 5	Cytoplasm	enzyme
NM_148962.4	OXER1	oxoeicosanoid (OXE) receptor 1	Plasma Membrane	G-protein coupled receptor
NM_000430.3	PAFAH1B1	platelet activating factor acetylhydrolase 1b regulatory subunit 1	Cytoplasm	enzyme
NM_016297.3	PCYOX1	prenylcysteine oxidase 1	Cytoplasm	enzyme
NM_002599.3	PDE2A	phosphodiesterase 2A	Cytoplasm	enzyme
NR_028444.1	PDIA5	protein disulfide isomerase family A member 5	Cytoplasm	enzyme
NM_001029891.2	PGAM4	phosphoglycerate mutase family member 4	Cytoplasm	phosphatase
NM_012396.3	PHLDA3	pleckstrin homology-like domain, family A, member 3	Plasma Membrane	other
NM_015993.2	PLLP	plasmolipin	Plasma Membrane	transporter
NM_003713.3	PLPP3	phospholipid phosphatase 3	Plasma Membrane	phosphatase
NM_015103.2	PLXND1	plexin D1	Plasma Membrane	transmembrane receptor
NM_006231.2	POLE	polymerase (DNA directed), epsilon, catalytic subunit	Nucleus	enzyme
NR_027390.1	POLR2M	polymerase (RNA) II (DNA directed) polypeptide M	Nucleus	other
NM_001018161.1	PON2	paraoxonase 2	Plasma Membrane	enzyme
NM_004575.2	POU4F2	POU class 4 homeobox 2	Nucleus	transcription regulator
NM_000942.4	PPIB	peptidylprolyl isomerase B	Cytoplasm	enzyme
NM_032902.5	PPP1R16A	protein phosphatase 1 regulatory subunit 16A	Plasma Membrane	other
NM_002574.2	PRDX1	peroxiredoxin 1	Cytoplasm	enzyme
NM_014098.2	PRDX3	peroxiredoxin 3	Cytoplasm	enzyme
NM_002734.3	PRKAR1A	protein kinase, cAMP-dependent, regulatory subunit type I alpha	Cytoplasm	kinase
NM_002764.3	PRPS1	phosphoribosyl pyrophosphate synthetase 1	Cytoplasm	kinase
NM_000314.4	PTEN	phosphatase and tensin homolog	Cytoplasm	phosphatase
NM_012232.5	PTRF	polymerase I and transcript release factor	Nucleus	transcription regulator
NM_138774.3	R3HDM4	R3H domain containing 4	Other	other
NM_014999.2	RAB21	RAB21, member RAS oncogene family	Cytoplasm	enzyme
NM_001142624.2	RAB34	RAB34, member RAS oncogene family	Cytoplasm	enzyme
NM_006861.6	RAB35	RAB35, member RAS oncogene family	Cytoplasm	enzyme
NM_001031834.1	RAB40AL	RAB40A, member RAS oncogene family-like	Plasma Membrane	other
NM_012415.2	RAD54B	RAD54 homolog B (S. cerevisiae)	Nucleus	enzyme

NM_015646.4	RAP1B	RAP1B, member of RAS oncogene family	Cytoplasm	enzyme
NM_006910.4	RBBP6	retinoblastoma binding protein 6	Nucleus	enzyme
NM_005822.2	RCAN2	regulator of calcineurin 2	Other	other
NM_020663.3	RHOJ	ras homolog family member J	Cytoplasm	enzyme
NM_022780.3	RMND5A	required for meiotic nuclear division 5 homolog A	Nucleus	other
NM_018434.4	RNF130	ring finger protein 130	Cytoplasm	peptidase
NM_001010858.2	RNF187	ring finger protein 187	Nucleus	enzyme
NM_000988.3	RPL27	ribosomal protein L27	Cytoplasm	other
NM_001000.2	RPL39	ribosomal protein L39	Cytoplasm	other
NM_001025.4	RPS23	ribosomal protein S23	Cytoplasm	translation regulator
NM_001135592.2	RPS27A	ribosomal protein S27a	Cytoplasm	other
NM_015920.3	RPS27L	ribosomal protein S27 like	Cytoplasm	translation regulator
NM_001006.3	RPS3A	ribosomal protein S3A	Nucleus	other
NM_152682.2	RWDD4	RWD domain containing 4	Other	other
NM_005979.2	S100A13	S100 calcium binding protein A13	Cytoplasm	other
NM_002970.2	SAT1	spermidine/spermine N1-acetyltransferase 1	Cytoplasm	enzyme
NM_006918.4	SC5D	sterol-C5-desaturase	Cytoplasm	enzyme
NM_004719.2	SCAF11	SR-related CTD-associated factor 11	Nucleus	other
NM_173833.4	SCARA5	scavenger receptor class A member 5	Cytoplasm	other
NM_001037582.2	SCD5	stearoyl-CoA desaturase 5	Cytoplasm	enzyme
NM_002979.3	SCP2	sterol carrier protein 2	Cytoplasm	transporter
NM_014300.2	SEC11A	SEC11 homolog A, signal peptidase complex subunit	Cytoplasm	peptidase
NM_001093726.1	SEPP1	selenoprotein P, plasma, 1	Extracellular Space	other
NM_004155.4	SERPINB9	serpin peptidase inhibitor, clade B (ovalbumin), member 9	Cytoplasm	other
NM_053282.4	SH2D1B	SH2 domain containing 1B	Cytoplasm	other
NM_001017995.2	SH3PXD2B	SH3 and PX domains 2B	Cytoplasm	other
NM_001146281.1	SHBG	sex hormone-binding globulin	Extracellular Space	other
NM_001006641.1	SLC25A25	solute carrier family 25 (mitochondrial carrier; phosphate carrier), member 25	Cytoplasm	transporter
NM_001152.4	SLC25A5	solute carrier family 25 (mitochondrial carrier; adenine nucleotide translocator), member 5	Cytoplasm	transporter
NM_003039.2	SLC2A5	solute carrier family 2 (facilitated glucose/fructose transporter), member 5	Plasma Membrane	transporter
NM_007001.2	SLC35D2	solute carrier family 35 (UDP-GlcNAc/UDP-glucose transporter), member D2	Cytoplasm	transporter
NM_152462.2	SLC35G3	solute carrier family 35 member G3	Other	enzyme
NM_018976.4	SLC38A2	solute carrier family 38 member 2	Plasma Membrane	transporter
NM_001135919.1	SLC46A3	solute carrier family 46 member 3	Extracellular Space	other
NM_018121.3	SLF2	SMC5-SMC6 complex localization factor 2	Extracellular Space	other
NM_007159.2	SLMAP	sarcolemma associated protein	Plasma Membrane	other
NM_001145432.1	SMIM20	small integral membrane protein 20	Other	other
NM_001124767.1	SMIM4	small integral membrane protein 4	Cytoplasm	other
NM_013306.3	SNX15	sorting nexin 15	Cytoplasm	transporter

NM_003877.3	SOCS2	suppressor of cytokine signaling 2	Cytoplasm	other
NM_003955.3	SOCS3	suppressor of cytokine signaling 3	Cytoplasm	phosphatase
NM_000454.4	SOD1	superoxide dismutase 1, soluble	Cytoplasm	enzyme
NM_001003845.2	SP5	Sp5 transcription factor	Nucleus	other
NM_004684.4	SPARCL1	SPARC like 1	Extracellular Space	other
NM_178313.2	SPTBN1	spectrin beta, non-erythrocytic 1	Plasma Membrane	other
NM_001013694.2	SRRD	SRR1 domain containing	Other	other
NM_006925.3	SRSF5	serine/arginine-rich splicing factor 5	Nucleus	other
NM_024636.2	STEAP4	STEAP4 metalloreductase	Plasma Membrane	enzyme
NM_004099.4	STOM	stomatin	Plasma Membrane	other
NM_003569.2	STX7	syntaxin 7	Plasma Membrane	transporter
NM_031914.2	SYT16	synaptotagmin 16	Other	transporter
NM_003967.2	TAAR5	trace amine associated receptor 5	Plasma Membrane	G-protein coupled receptor
NM_004607.2	TBCA	tubulin folding cofactor A	Cytoplasm	other
NM_153333.2	TCEAL8	transcription elongation factor A (SII)-like 8	Other	other
NM_153035.1	TCEANC2	transcription elongation factor A (SII) N-terminal and central domain containing 2	Other	other
NM_138501.4	TECR	trans-2,3-enoyl-CoA reductase	Plasma Membrane	enzyme
NM_016020.2	TFB1M	transcription factor B1, mitochondrial	Cytoplasm	transcription regulator
NM_001024847.2	TGFBR2	transforming growth factor beta receptor II	Plasma Membrane	kinase
NR_028383.1	TIMM8B	translocase of inner mitochondrial membrane 8 homolog B (yeast)	Cytoplasm	transporter
NM_000362.4	TIMP3	TIMP metallopeptidase inhibitor 3	Extracellular Space	other
NM_014742.3	TM9SF4	transmembrane 9 superfamily protein member 4	Cytoplasm	transporter
NM_152468.4	TMC8	transmembrane channel like 8	Cytoplasm	other
NR_002807.3	TMED10P1	transmembrane emp24-like trafficking protein 10 (yeast) pseudogene 1	Other	other
NM_032323.2	TMEM79	transmembrane protein 79	Cytoplasm	other
NM_006290.2	TNFAIP3	TNF alpha induced protein 3	Nucleus	enzyme
NM_003842.4	TNFRSF10B	tumor necrosis factor receptor superfamily member 10b	Plasma Membrane	transmembrane receptor
NM_022748.10	TNS3	tensin 3	Plasma Membrane	phosphatase
NM_001037330.1	TRIM16L	tripartite motif containing 16-like	Other	other
NM_016113.3	TRPV2	transient receptor potential cation channel, subfamily V, member 2	Plasma Membrane	ion channel
NM_004089.3	TSC22D3	TSC22 domain family member 3	Nucleus	transcription regulator
NM_030935.3	TSC22D4	TSC22 domain family member 4	Nucleus	transcription regulator
NM_173485.4	TSHZ2	teashirt zinc finger homeobox 2	Other	other
NM_005723.3	TSPAN5	tetraspanin 5	Plasma Membrane	other
NR_001545.2	TTTY15	testis-specific transcript, Y-linked 15 (non-protein coding)	Other	other

NM_006009.2	TUBA1A	tubulin alpha 1a	Cytoplasm	other
NM_172070.3	UBR3	ubiquitin protein ligase E3 component n-recognin 3 (putative)	Other	enzyme
NM_080911.1	UNG	uracil DNA glycosylase	Nucleus	enzyme
NM_004654.3	USP9Y	ubiquitin specific peptidase 9, Y-linked	Cytoplasm	peptidase
NM_003374.1	VDAC1	voltage dependent anion channel 1	Cytoplasm	ion channel
NM_030938.3	VMP1	vacuole membrane protein 1	Plasma Membrane	other
NM_006646.5	WASF3	WAS protein family member 3	Cytoplasm	other
NM_003881.2	WISP2	WNT1 inducible signaling pathway protein 2	Extracellular Space	growth factor
NM_014263.2	YME1L1	YME1 like 1 ATPase	Cytoplasm	peptidase
NM_024784.3	ZBTB3	zinc finger and BTB domain containing 3	Nucleus	other
NM_003407.2	ZFP36	ZFP36 ring finger protein	Nucleus	transcription regulator
NM_173832.3	ZFP41	ZFP41 zinc finger protein	Nucleus	other
NM_024620.3	ZNF329	zinc finger protein 329	Other	other
NM_207341.2	ZP1	zona pellucida glycoprotein 1	Extracellular Space	other
XM_002346324.1		unknown	unknown	unknown
XR_038342.2		unknown	unknown	unknown
XR_078642.1		unknown	unknown	unknown
XR_079072.1		unknown	unknown	unknown

Supplementary Table S3. Genes up-regulated in Cluster B involved in cell death

**Categories**	**Functions Annotation**	**p-Value**	**Activation z-score**	**Molecules**
Cell Death and Survival	apoptosis of tumor cell lines	8,69E-11	-1,003	ACVR1B,ATF3,B2M,BCL2,BTG2,CAV1,CCND3,CD99,CDC42,CEBPD,COX8A,CYB5A,DAPK3,DUSP1,EBF1,EGR1,EIF2A,ETS1,ETS2,FN1,FOSB,GJA1,GPX1,GSN,GSTA2,HSPD1,IER3,IL1R1,INHBA,JUN,JUND,KLF2,MAX,NCAM1,NDRG2,NFE2L1,OGT,PRDX1,PRKAR1A,PRPS1,PTEN,RBBP6,RHOJ,SAT1,SLC25A5,SOCS3,SOD1,TGFBR2,TIMP3,TNFAIP3,TNFRSF10B,TSC22D3,TUBA1A,VDAC1
Cell Death and Survival	cell death	1,34E-09	-1,115	ACVR1B,AK1,ALDH2,ALDOA,ANXA5,ARL6IP5,ATF3,B2M,BCL2,BSG,BTG2,CAV1,CCND3,CCT5,CD6,CD99,CDC42,CEBPD,CFB,CFI,CHMP3,COL5A3,COX8A,CSTB,CYB5A,CYP11A1,DAPK3,DLK1,DPP9,DUSP1,DUSP2,EBF1,EGLN1,EGR1,EIF2A,ETS1,ETS2,FEN1,FN1,FOSB,FUS,GJA1,GPX1,GSN,GSTA2,HERPUD1,HLA-A,HSPD1,IER3,IL1R1,INHBA,JUN,JUND,KLF2,LDLR,LY9,MAP1B,MAX,MGAT5,NCAM1,NCOR2,NDRG2,NFE2L1,NGFRAP1,NSMAF,OGT,PAFAH1B1,PHLDA3,PON2,POU4F2,PPIB,PRDX1,PRDX3,PRKAR1A,PRPS1,PTEN,RAB35,RAP1B,RBBP6,RCAN2,RHOJ,RNF130,RPS3A,SAT1,SCP2,SEPP1,SERPINB9,SH2D1B,SHBG,SLC25A5,SOCS2,SOCS3,SOD1,SPTBN1,STOM,TFB1M,TGFBR2,TIMP3,TNFAIP3,TNFRSF10B,TSC22D3,TUBA1A,UNG,VDAC1,ZFP36
Cell Death and Survival	apoptosis	1,88E-09	-1,577	ACVR1B,ALDH2,ALDOA,ANXA5,ARL6IP5,ATF3,B2M,BCL2,BSG,BTG2,CAV1,CCND3,CD99,CDC42,CEBPD,COL5A3,COX8A,CSTB,CYB5A,CYP11A1,DAPK3,DPP9,DUSP1,DUSP2,EBF1,EGR1,EIF2A,ETS1,ETS2,FEN1,FN1,FOSB,FUS,GJA1,GPX1,GSN,GSTA2,HERPUD1,HSPD1,IER3,IL1R1,INHBA,JUN,JUND,KLF2,LDLR,MAP1B,MAX,NCAM1,NCOR2,NDRG2,NFE2L1,NGFRAP1,NSMAF,OGT,PAFAH1B1,PHLDA3,PON2,PRDX1,PRDX3,PRKAR1A,PRPS1,PTEN,RAP1B,RBBP6,RCAN2,RHOJ,RNF130,RPS3A,SAT1,SEPP1,SERPINB9,SHBG,SLC25A5,SOCS2,SOCS3,SOD1,SPTBN1,TFB1M,TGFBR2,TIMP3,TNFAIP3,TNFRSF10B,TSC22D3,TUBA1

				A,UNG,VDAC1,ZFP36
Cell Death and Survival	cell death of tumor cell lines	2,05E-09	-1,876	ACVR1B,ATF3,B2M,BCL2,BTG2,CAV1,CCND3,CCT5,CD99,CDC42,CEBPD,COX8A,CYB5A,DAPK3,DPP9,DUSP1,EBF1,EGLN1,EGR1,EIF2A,ETS1,ETS2,FEN1,FN1,FOSB,GJA1,GPX1,GSN,GSTA2,HERPUD1,HSPD1,IER3,IL1R1,INHBA,JUN,JUND,KLF2,MAX,NCAM1,NDRG2,NFE2L1,OGT,PRDX1,PRKAR1A,PRPS1,PTEN,RAB35,RBBP6,RHOJ,SAT1,SLC25A5,SOCS3,SOD1,TGFBR2,TIMP3,TNFAIP3,TNFRSF10B,TSC22D3,TUBA1A,VDAC1
Cell Death and Survival	necrosis	4,29E-09	-1,553	ACVR1B,ALDH2,ALDOA,ATF3,B2M,BCL2,BSG,BTG2,CAV1,CCND3,CCT5,CD99,CDC42,CEBPD,COL5A3,COX8A,CYB5A,CYP11A1,DAPK3,DLK1,DPP9,DUSP1,EBF1,EGLN1,EGR1,EIF2A,ETS1,ETS2,FEN1,FN1,FOSB,FUS,GJA1,GPX1,GSN,GSTA2,HERPUD1,HSPD1,IER3,IL1R1,INHBA,JUN,JUND,KLF2,LDLR,MAP1B,MAX,MGAT5,NCAM1,NCOR2,NDRG2,NFE2L1,NGFRAP1,NSMAF,OGT,PAFAH1B1,PON2,POU4F2,PRDX1,PRDX3,PRKAR1A,PRPS1,PTEN,RAB35,RBBP6,RCAN2,RHOJ,RPS3A,SAT1,SCP2,SEPP1,SERPINB9,SLC25A5,SOCS3,SOD1,SPTBN1,TFB1M,TGFBR2,TIMP3,TNFAIP3,TNFRSF10B,TSC22D3,TUBA1A,UNG,VDAC1,ZFP36
Cell Death and Survival	cytolysis	3,55E-07	-1,628	AK1,ALDOA,B2M,BCL2,CAV1,CFB,CFI,ETS1,GJA1,GPX1,HLA-A,LY9,PRDX1,SERPINB9,SH2D1B,STOM,TGFBR2,TNFRSF10B
Cell Death and Survival	necrosis of epithelial tissue	1,09E-06	-0,084	ALDH2,ATF3,BCL2,CAV1,CDC42,CEBPD,EGR1,GPX1,HSPD1,IER3,INHBA,JUN,KLF2,LDLR,MAX,NDRG2,PRDX3,PTEN,SEPP1,SLC25A5,SOCS3,SOD1,SPTBN1,TGFBR2,TIMP3,TNFAIP3,TNFRSF10B,VDAC1
Cell Death and Survival	apoptosis of leukemia cell lines	1,97E-06	-0,351	B2M,BCL2,CAV1,CEBPD,DUSP1,EIF2A,GSN,GSTA2,INHBA,JUN,MAX,OGT,PTEN,SOCS3,TNFAIP3,TNFRSF10B,TSC22D3
Cell Death and Survival	apoptosis of prostate cancer cell lines	2,34E-06	-0,004	BCL2,CAV1,EGR1,ETS2,FN1,HSPD1,INHBA,JUN,PRKAR1A,PTEN,SLC25A5,SOCS3,TNFRSF10B
Cell Death and Survival	cell death of leukemia cell lines	3,22E-06	-0,550	B2M,BCL2,CAV1,CD99,CEBPD,DUSP1,EIF2A,GSN,GSTA2,INHBA,JUN,MAX,OGT,PTEN,SOCS3,TNFAIP3,TNFRSF10B,TSC22D3
Cell Death and Survival, Hair and Skin Development and Function	cell viability of epithelial cell lines	2,00E-05	2,158	BCL2,BTG2,CALB2,CAV1,CDC42,CEBPD,JUN,TNFAIP3
Cell Death and Survival, Gastrointestinal Disease, Hepatic System Disease	necrosis of liver	2,43E-05	-1,663	BCL2,HSPD1,IER3,INHBA,JUN,JUND,LDLR,NFE2L1,SEPP1,SLC25A5,SOCS3,SOD1,SPTBN1,TIMP3
Cell Death and Survival	cell death of epithelial cells	3,39E-05	1,502	ATF3,BCL2,CDC42,CEBPD,EGR1,HSPD1,IER3,INHBA,JUN,LDLR,NDRG2,PRDX3,PTEN,SEPP1,SLC25A5,SOCS3,SOD1,SPTBN1,TGFBR2,TIMP3,TNFRSF10B,VDAC1
Cell Death and Survival	anoikis of tumor cell lines	3,73E-05	0,714	BCL2,CAV1,CD99,FN1,PTEN,TNFRSF10B
Cell Death and Survival	cell death of cortical neurons	4,24E-05	0,558	BCL2,CDC42,DUSP1,ETS2,FUS,HSPD1,IL1R1,JUN,MAP1B,NGFRAP1,UNG
Cancer, Cell Death and Survival, Tumor Morphology	cell death of tumor cells	4,24E-05	-0,042	B2M,BCL2,BSG,CAV1,CCND3,ETS1,INHBA,JUN,NCOR2,PON2,PTEN,RPS3A,SERPINB9,SOCS3,SOD1,TGFBR2,TNFRSF10B,ZFP36
Cell Death and Survival, Gastrointestinal Disease, Hepatic System Disease	cell death of liver cells	4,40E-05	-1,229	BCL2,HSPD1,IER3,INHBA,JUN,JUND,LDLR,SEPP1,SLC25A5,SOCS3,SPTBN1,TIMP3
Cell Death and Survival	cell survival	4,84E-05	4,749	ATF3,BCL2,BTG2,CALB2,CARD10,CAV1,CDC42,CDH13,CEBPD,DLK1,DPP9,DRG2,DUSP1,EGR1,ETS2,FN1,GJA1,GPX1,HERPUD1,HSPD1,JUN,JUND,KLF2,MGAT5,MTMR7,NCAM1,NDRG2,NGFRAP1,OGT,POU4F2,PPIB,PRKAR1A,PTEN,RHOJ,RPL27,SERPINB9,SOCS2,SOCS3,SOD1,TGFBR2,TNFAIP3,TNFRSF10B,UNG,VDAC1,ZFP36

Cell Death and Survival	cell death of T lymphocytes	6,58E-05	-1,384	BCL2,CAV1,CD99,CDC42,EGR1,ETS1,ETS2,IER3,JUN,KLF2,OGT,PTEN,RCAN2,SERPINB9,TGFBR2,TSC22D3
Cell Death and Survival	apoptosis of vascular endothelial cells	7,08E-05	-0,705	ALDH2,ATF3,BCL2,CAV1,HSPD1,PTEN,TNFRSF10B,VDAC1
Cell Death and Survival	cell death of endothelial cells	7,75E-05	-1,753	ALDH2,ATF3,BCL2,CAV1,GPX1,HSPD1,KLF2,MAX,PTEN,TNFAIP3,TNFRSF10B,VDAC1
Cell Death and Survival	killing of splenocytes	8,58E-05		BCL2,ETS1,SH2D1B
Cell Death and Survival	cell death of brain cells	9,70E-05	0,501	BCL2,CDC42,DUSP1,EGR1,ETS2,FUS,HSPD1,IL1R1,JUN,MAP1B,NGFRAP1,PTEN,SOD1,UNG
Cell Death and Survival	neuronal cell death	1,08E-04	-0,674	ATF3,BCL2,BTG2,CDC42,DUSP1,EGLN1,EGR1,ETS2,FN1,FUS,GJA1,GPX1,HERPUD1,HSPD1,IL1R1,INHBA,JUN,MAP1B,NGFRAP1,PAFAH1B1,POU4F2,PRDX3,PTEN,SOD1,TFB1M,UNG
Cell Death and Survival	apoptosis of sympathetic neuron	1,09E-04	-0,818	BCL2,CDC42,DUSP1,JUN,SOD1
Cell Death and Survival	apoptosis of liver	1,24E-04	-1,627	BCL2,INHBA,JUN,JUND,LDLR,NFE2L1,SEPP1,SOCS3,SPTBN1,TIMP3
Cell Death and Survival	apoptosis of endothelial cells	1,30E-04	-1,554	ALDH2,ATF3,BCL2,CAV1,GPX1,HSPD1,MAX,PTEN,TNFAIP3,TNFRSF10B,VDAC1
Cell Death and Survival	cell death of fibroblasts	1,44E-04	-0,186	ATF3,BCL2,CDC42,DUSP1,GPX1,JUN,JUND,KLF2,NSMAF,PTEN,TIMP3,TNFRSF10B,UNG
Cell Death and Survival	cell death of carcinoma cell lines	1,53E-04	-0,081	BCL2,DAPK3,DUSP1,ETS2,GJA1,GPX1,JUN,KLF2,OGT,PRKAR1A,SOD1,TNFRSF10B
Cell Death and Survival	cell death of cerebral cortex cells	1,65E-04	0,432	BCL2,CDC42,DUSP1,ETS2,FUS,HSPD1,IL1R1,JUN,MAP1B,NGFRAP1,SOD1,UNG
Cell Death and Survival	cell death of myeloma cell lines	1,81E-04	-0,178	B2M,BCL2,INHBA,JUN,NCAM1,PTEN,TNFRSF10B
Cell Death and Survival	cell death of pre-oligodendrocytes	1,88E-04		BCL2,SOD1
Cell Death and Survival	apoptosis of T lymphocytes	1,93E-04	-1,326	BCL2,CAV1,CDC42,EGR1,ETS1,ETS2,IER3,JUN,KLF2,OGT,PTEN,RCAN2,SERPINB9,TSC22D3
Cell Death and Survival, Connective Tissue Disorders, Hematological Disease	hemolytic anemia	1,94E-04		AK1,ALDOA,CFB,CFI,GPX1,PRDX1,STOM
Cell Death and Survival	cell death of immune cells	2,00E-04	0,004	ATF3,BCL2,CAV1,CD99,CDC42,DLK1,EGR1,ETS1,ETS2,FN1,FUS,GSN,IER3,INHBA,JUN,KLF2,LDLR,MGAT5,OGT,PTEN,RCAN2,SERPINB9,TGFBR2,TNFAIP3,TSC22D3
Cell Death and Survival	apoptosis of fibroblasts	2,40E-04	0,280	ATF3,BCL2,CDC42,DUSP1,JUN,JUND,KLF2,NSMAF,PTEN,TIMP3,TNFRSF10B
Cell Death and Survival	cell death of connective tissue cells	2,45E-04	-0,219	ATF3,BCL2,CDC42,DUSP1,EGR1,EIF2A,ETS1,FN1,FUS,GPX1,GSN,HSPD1,IER3,JUN,JUND,KLF2,NSMAF,PTEN,SCP2,TIMP3,TNFAIP3,TNFRSF10B,UNG
Cell Death and Survival	apoptosis of pheochromocytoma cell lines	2,57E-04	-1,638	ATF3,BCL2,BTG2,CDC42,IL1R1,JUN,SOD1
Cell Death and Survival	cell death of bone cancer cell lines	2,57E-04	0,454	BCL2,CAV1,CD99,DPP9,ETS1,OGT,PRDX1,PRPS1,RBBP6,TNFRSF10B
Cell Death and Survival	cell viability	2,60E-04	4,439	BCL2,BTG2,CALB2,CARD10,CAV1,CDC42,CDH13,CEBPD,DLK1,DPP9,DRG2,DUSP1,EGR1,ETS2,FN1,GJA1,GPX1,HERPUD1,HSPD1,JUN,KLF2,MGAT5,MTMR7,NCAM1,OGT,POU4F2,PRKAR1A,PTEN,RHOJ,RPL27,SERPINB9,SOCS2,SOCS3,SOD1,TGFBR2,TNFAIP3,TNFRSF10B,UNG,VDAC1,ZFP36
Cell Death and Survival	apoptosis of melanoma cell lines	2,67E-04	-1,212	BCL2,EGR1,ETS1,JUN,PRDX1,RHOJ,SAT1,TNFRSF10B
Cell Death and Survival	cell death of lymphoma cell lines	3,02E-04	-1,239	BCL2,BTG2,CCND3,CD99,DUSP1,EBF1,EGR1,GPX1,JUN,MAX,TNFRSF10B

Cell Death and Survival	apoptosis of brain	3,41E-04	0,111	BCL2,CDC42,DUSP1,EGR1,JUN,MAP1B,PTEN,SOD1,UNG
Cell Death and Survival	cytotoxicity	3,48E-04	-0,084	B2M,BCL2,CD6,FN1,HLA-A,IL1R1,JUN,LDLR,PPIB,SOD1,TGFBR2,TNFAIP3
Cell Death and Survival	cell death of pheochromocytoma cell lines	3,52E-04	-2,000	ATF3,BCL2,BTG2,CDC42,HERPUD1,IL1R1,JUN,SOD1
Cell Death and Survival, Renal and Urological System Development and Function	cell viability of kidney cell lines	3,67E-04	0,128	BCL2,CAV1,CEBPD,PTEN,TNFAIP3,VDAC1
Cell Death and Survival	cell death of muscle cells	4,33E-04	-1,070	ALDOA,BCL2,CAV1,ETS1,GPX1,GSN,HSPD1,INHBA,JUND,PRDX3,PTEN,SOCS3,TIMP3,TNFAIP3,TNFRSF10B
Cell Death and Survival	apoptosis of carcinoma cell lines	4,40E-04	0,291	BCL2,DAPK3,DUSP1,ETS2,GJA1,GPX1,KLF2,OGT,PRKAR1A,TNFRSF10B
Cell Death and Survival, Gastrointestinal Disease, Hepatic System Disease	cell death of hepatocytes	4,69E-04	-0,958	BCL2,INHBA,JUN,LDLR,SEPP1,SLC25A5,SOCS3,SPTBN1,TIMP3
Cell Death and Survival, Gastrointestinal Disease, Hepatic System Disease	apoptosis of liver cells	5,11E-04	-1,407	BCL2,INHBA,JUN,JUND,LDLR,SEPP1,SOCS3,SPTBN1,TIMP3
Cell Death and Survival, Hematological System Development and Function	survival of pre-T lymphocytes	5,58E-04		BCL2,DLK1
Cell Death and Survival	apoptosis of myeloma cell lines	5,75E-04	-0,577	B2M,BCL2,JUN,NCAM1,PTEN,TNFRSF10B
Cancer, Cell Death and Survival, Tumor Morphology	apoptosis of tumor cells	6,54E-04	0,085	B2M,BCL2,BSG,CAV1,CCND3,ETS1,JUN,NCOR2,PON2,PTEN,SOCS3,TGFBR2,TNFRSF10B
Cell Death and Survival, Gastrointestinal Disease, Hepatic System Disease	apoptosis of hepatocytes	6,77E-04	-0,958	BCL2,INHBA,JUN,LDLR,SEPP1,SOCS3,SPTBN1,TIMP3
Cancer, Cell Death and Survival, Tumor Morphology	apoptosis of melanoma cells	7,17E-04		BCL2,ETS1,JUN,TNFRSF10B
Cell Death and Survival	apoptosis of neurons	7,18E-04	-1,571	ATF3,BCL2,BTG2,CDC42,DUSP1,EGR1,FN1,GPX1,HERPUD1,HSPD1,JUN,MAP1B,PAFAH1B1,PRDX3,SOD1,TFB1M,UNG
Cell Death and Survival, Cellular Compromise	toxicity of cells	7,29E-04	-0,270	B2M,BCL2,CD6,FN1,HLA-A,IL1R1,JUN,LDLR,SOD1,TGFBR2,TNFAIP3
Cell Death and Survival	apoptosis of leukocytes	7,36E-04	-1,115	ATF3,BCL2,CAV1,CDC42,EGR1,ETS1,ETS2,GSN,IER3,JUN,KLF2,LDLR,OGT,PTEN,RCAN2,SERPINB9,TNFAIP3,TSC22D3
Cell Death and Survival	cell death of endothelial cell lines	7,85E-04	-2,236	BCL2,FN1,GPX1,SOD1,TNFRSF10B
Cell Death and Survival	apoptosis of fibroblast cell lines	8,55E-04	0,258	ATF3,BCL2,EGR1,EIF2A,ETS1,FN1,FUS,GSN,HSPD1,IER3,JUN,PTEN,TNFAIP3,TNFRSF10B

Cell Death and Survival	apoptosis of muscle cells	1,03E-03	-0,812	ALDOA,BCL2,GPX1,GSN,HSPD1,INHBA,JUND,PRDX3,PTEN,SOCS3,TIMP3,TNFAIP3
Cell Death and Survival	apoptosis of brain cells	1,07E-03	0,399	BCL2,CDC42,EGR1,JUN,MAP1B,PTEN,SOD1,UNG
Cell Death and Survival	apoptosis of bone cancer cell lines	1,16E-03	0,747	BCL2,CAV1,CD99,ETS1,OGT,PRPS1,RBBP6,TNFRSF10B
Cell Death and Survival	apoptosis of smooth muscle cell lines	1,25E-03		BCL2,ETS1,TNFRSF10B
Cell Death and Survival	cell death of ganglion cells	1,30E-03		ATF3,BCL2,SOD1,TFB1M
Cancer, Cell Death and Survival, Tumor Morphology	cell death of mammary tumor cells	1,30E-03		BCL2,CAV1,CCND3,JUN
Cancer, Cell Death and Survival, Tumor Morphology	apoptosis of lung cancer cells	1,51E-03		PTEN,SOCS3,TNFRSF10B
Cell Death and Survival	cell viability of stomach cancer cell lines	1,51E-03		BCL2,CAV1,PTEN
Cell Death and Survival	apoptosis of breast cancer cell lines	1,51E-03	-0,955	B2M,BCL2,CAV1,CYB5A,DUSP1,ETS2,FN1,HSPD1,IER3,JUN,PTEN,TNFRSF10B
Cell Death and Survival	cell viability of breast cancer cell lines	1,68E-03	2,752	BCL2,BTG2,CARD10,CEBPD,DUSP1,GPX1,JUN,PTEN
Cell Death and Survival	cellular degradation	1,71E-03	-1,425	BCL2,CAV1,CHMP3,ETS2,GPX1,JUN,NCAM1,POU4F2,SOD1,TGFBR2,UNG
Cell Death and Survival	cell death of epithelial cell lines	1,72E-03	1,242	ATF3,BCL2,CDC42,CEBPD,HSPD1,IER3,JUN,PRDX3,SOD1,TGFBR2,TNFRSF10B,VDAC1
Cell Death and Survival	apoptosis of connective tissue cells	1,81E-03	-0,052	ATF3,BCL2,CDC42,DUSP1,FN1,JUN,JUND,KLF2,NSMAF,PTEN,TIMP3,TNFRSF10B
Cancer, Cell Death and Survival, Tumor Morphology	apoptosis of non-small-cell lung cancer cells	1,83E-03		PTEN,SOCS3
Cell Death and Survival	apoptosis of epithelial cells	2,06E-03	0,088	BCL2,EGR1,INHBA,JUN,LDLR,NDRG2,PTEN,SEPP1,SOCS3,SPTBN1,TIMP3,TNFRSF10B
Cell Death and Survival, Reproductive System Development and Function	cell viability of breast cell lines	2,10E-03		BTG2,DUSP1,ETS2
Cell Death and Survival	cell death of breast cancer cell lines	2,12E-03	-1,113	B2M,BCL2,CAV1,CDC42,CYB5A,DUSP1,ETS2,FN1,HSPD1,IER3,JUN,PTEN,TNFRSF10B
Cell Death and Survival	apoptosis of epithelial cell lines	2,13E-03	0,971	ATF3,BCL2,CEBPD,HSPD1,IER3,JUN,PRDX3,TGFBR2,TNFRSF10B,VDAC1
Cell Death and Survival, Cellular Compromise	cytotoxicity of cells	2,19E-03	-0,503	B2M,BCL2,CD6,FN1,HLA-A,JUN,LDLR,SOD1,TGFBR2,TNFAIP3
Cell Death and Survival	cell death of hepatoma cell lines	2,20E-03	0,992	ACVR1B,BCL2,FOSB,INHBA,JUND,PTEN,SOD1,TNFRSF10B

Supplementary Table S4. Genes up-regulated in both oligospermic and azoospermic patients

ID	Symbol	Entrez Gene Name	Location	Type(s)
ABI3BP	ABI3BP	ABI family member 3 binding protein	Extracellular Space	other
ACAP1	ACAP1	ArfGAP with coiled-coil, ankyrin repeat and PH domains 1	Plasma Membrane	other
ACSF2	ACSF2	acyl-CoA synthetase family member 2	Cytoplasm	enzyme
ANKRD40	ANKRD40	ankyrin repeat domain 40	Other	other
ANXA5	ANXA5	annexin A5	Plasma Membrane	transporter
AOX1	AOX1	aldehyde oxidase 1	Cytoplasm	enzyme
ARL6IP5	ARL6IP5	ADP ribosylation factor like GTPase 6 interacting protein 5	Cytoplasm	other
ATP5C1	ATP5C1	ATP synthase, H+ transporting, mitochondrial F1 complex, gamma polypeptide 1	Cytoplasm	transporter
B2M	B2M	beta-2-microglobulin	Plasma Membrane	transmembrane receptor
BRD3	BRD3	bromodomain containing 3	Nucleus	kinase
BTBD3	BTBD3	BTB (POZ) domain containing 3	Other	other
BTG2	BTG2	BTG family member 2	Nucleus	transcription regulator
C2orf70	C2orf70	chromosome 2 open reading frame 70	Nucleus	other
CAPZA2	CAPZA2	capping protein (actin filament) muscle Z-line, alpha 2	Cytoplasm	other
CAV1	CAV1	caveolin 1	Plasma Membrane	transmembrane receptor
CD6	CD6	CD6 molecule	Plasma Membrane	transmembrane receptor
CD63	CD63	CD63 molecule	Plasma Membrane	other
CEBPD	CEBPD	CCAAT/enhancer binding protein delta	Nucleus	transcription regulator
CETN2	CETN2	centrin 2	Nucleus	enzyme
COX8A	COX8A	cytochrome c oxidase subunit VIIIA (ubiquitous)	Cytoplasm	enzyme
CSTB	CSTB	cystatin B	Cytoplasm	peptidase
CYB5A	CYB5A	cytochrome b5 type A (microsomal)	Cytoplasm	enzyme
DSTN	DSTN	destrin (actin depolymerizing factor)	Cytoplasm	other
DUSP1	DUSP1	dual specificity phosphatase 1	Nucleus	phosphatase
DUSP16	DUSP16	dual specificity phosphatase 16	Nucleus	phosphatase
DYNLRB1	DYNLRB1	dynein, light chain, roadblock-type 1	Cytoplasm	other
ETS1	ETS1	v-ets avian erythroblastosis virus E26 oncogene homolog 1	Nucleus	transcription regulator
FERMT2	FERMT2	fermitin family member 2	Cytoplasm	other
FOXC1	FOXC1	forkhead box C1	Nucleus	transcription regulator
FTH1P3	FTH1P3	ferritin, heavy polypeptide 1 pseudogene 3	Other	other
FTL	FTL	ferritin, light polypeptide	Cytoplasm	enzyme
FUS	FUS	FUS RNA binding protein	Nucleus	transcription regulator
GNB5	GNB5	guanine nucleotide binding protein (G protein), beta 5	Plasma Membrane	enzyme
GSN	GSN	gelsolin	Extracellular Space	other
GSTA2	GSTA2	glutathione S-transferase alpha 2	Cytoplasm	enzyme
GYPC	GYPC	glycophorin C (Gerbich blood group)	Plasma Membrane	other
HLA-DRA	HLA-DRA	major histocompatibility complex, class II, DR	Plasma	transmembrane

		alpha	Membrane	receptor
HOMER3	HOMER3	homer scaffolding protein 3	Plasma Membrane	other
HSD17B6	HSD17B6	hydroxysteroid (17-beta) dehydrogenase 6	Other	enzyme
INHBA	INHBA	inhibin beta A	Extracellular Space	growth factor
INO80D	INO80D	INO80 complex subunit D	Other	other
INS-IGF2	INS-IGF2	INS-IGF2 readthrough	Other	other
ITPRIPL2	ITPRIPL2	inositol 1,4,5-trisphosphate receptor interacting protein-like 2	Other	other
JUN	JUN	jun proto-oncogene	Nucleus	transcription regulator
KCTD12	KCTD12	potassium channel tetramerization domain containing 12	Plasma Membrane	ion channel
KLF2	KLF2	Kruppel-like factor 2	Nucleus	transcription regulator
LDLR	LDLR	low density lipoprotein receptor	Plasma Membrane	transporter
LSM14A	LSM14A	LSM14A mRNA processing body assembly factor	Cytoplasm	other
LY9	LY9	lymphocyte antigen 9	Plasma Membrane	other
MAMLD1	MAMLD1	mastermind like domain containing 1	Other	other
MAP1B	MAP1B	microtubule associated protein 1B	Cytoplasm	other
METTL7A	METTL7A	methyltransferase like 7A	Cytoplasm	other
MSMO1	MSMO1	methylsterol monooxygenase 1	Cytoplasm	enzyme
MYO1B	MYO1B	myosin IB	Cytoplasm	other
NCAM1	NCAM1	neural cell adhesion molecule 1	Plasma Membrane	other
NCOA7	NCOA7	nuclear receptor coactivator 7	Nucleus	transcription regulator
NDRG2	NDRG2	NDRG family member 2	Cytoplasm	other
NFIA	NFIA	nuclear factor I/A	Nucleus	transcription regulator
NGFRAP1	NGFRAP1	nerve growth factor receptor (TNFRSF16) associated protein 1	Cytoplasm	other
NPR2	NPR2	natriuretic peptide receptor 2	Plasma Membrane	G-protein coupled receptor
NSMAF	NSMAF	neutral sphingomyelinase activation associated factor	Cytoplasm	other
NXF1	NXF1	nuclear RNA export factor 1	Nucleus	transporter
OGN	OGN	osteoglycin	Extracellular Space	growth factor
OGT	OGT	O-linked N-acetylglucosamine (GlcNAc) transferase	Cytoplasm	enzyme
OTUD5	OTUD5	OTU deubiquitinase 5	Cytoplasm	enzyme
PAFAH1B1	PAFAH1B1	platelet activating factor acetylhydrolase 1b regulatory subunit 1	Cytoplasm	enzyme
PCYOX1	PCYOX1	prenylcysteine oxidase 1	Cytoplasm	enzyme
PLLP	PLLP	plasmolipin	Plasma Membrane	transporter
PRDX1	PRDX1	peroxiredoxin 1	Cytoplasm	enzyme
PRDX3	PRDX3	peroxiredoxin 3	Cytoplasm	enzyme
PRKAR1A	PRKAR1A	protein kinase, cAMP-dependent, regulatory subunit type I alpha	Cytoplasm	kinase
PTEN	PTEN	phosphatase and tensin homolog	Cytoplasm	phosphatase
RAB34	RAB34	RAB34, member RAS oncogene family	Cytoplasm	enzyme
RAP1B	RAP1B	RAP1B, member of RAS oncogene family	Cytoplasm	enzyme
RMND5A	RMND5A	required for meiotic nuclear division 5 homolog A	Nucleus	other

RNF130	RNF130	ring finger protein 130	Cytoplasm	peptidase
RPL27	RPL27	ribosomal protein L27	Cytoplasm	other
RPL39	RPL39	ribosomal protein L39	Cytoplasm	other
RPS23	RPS23	ribosomal protein S23	Cytoplasm	translation regulator
RPS27A	RPS27A	ribosomal protein S27a	Cytoplasm	other
RWDD4	RWDD4	RWD domain containing 4	Other	other
SAT1	SAT1	spermidine/spermine N1-acetyltransferase 1	Cytoplasm	enzyme
SC5D	SC5D	sterol-C5-desaturase	Cytoplasm	enzyme
SEC11A	SEC11A	SEC11 homolog A, signal peptidase complex subunit	Cytoplasm	peptidase
SH3PXD2B	SH3PXD2B	SH3 and PX domains 2B	Cytoplasm	other
SLC25A25	SLC25A25	solute carrier family 25 (mitochondrial carrier; phosphate carrier), member 25	Cytoplasm	transporter
SLC35D2	SLC35D2	solute carrier family 35 (UDP-GlcNAc/UDP-glucose transporter), member D2	Cytoplasm	transporter
SLC35G3	SLC35G3	solute carrier family 35 member G3	Other	enzyme
SLC38A2	SLC38A2	solute carrier family 38 member 2	Plasma Membrane	transporter
FAM178A	SLF2	SMC5-SMC6 complex localization factor 2	Extracellular Space	other
SMIM4	SMIM4	small integral membrane protein 4	Cytoplasm	other
SNX15	SNX15	sorting nexin 15	Cytoplasm	transporter
SOCS2	SOCS2	suppressor of cytokine signaling 2	Cytoplasm	other
SOCS3	SOCS3	suppressor of cytokine signaling 3	Cytoplasm	phosphatase
SOD1	SOD1	superoxide dismutase 1, soluble	Cytoplasm	enzyme
SPARCL1	SPARCL1	SPARC like 1	Extracellular Space	other
SPTBN1	SPTBN1	spectrin beta, non-erythrocytic 1	Plasma Membrane	other
SRSF5	SRSF5	serine/arginine-rich splicing factor 5	Nucleus	other
TECR	TECR	trans-2,3-enoyl-CoA reductase	Plasma Membrane	enzyme
TFB1M	TFB1M	transcription factor B1, mitochondrial	Cytoplasm	transcription regulator
TIMP3	TIMP3	TIMP metallopeptidase inhibitor 3	Extracellular Space	other
TNFAIP3	TNFAIP3	TNF alpha induced protein 3	Nucleus	enzyme
TNFRSF10B	TNFRSF10B	tumor necrosis factor receptor superfamily member 10b	Plasma Membrane	transmembrane receptor
TSC22D3	TSC22D3	TSC22 domain family member 3	Nucleus	transcription regulator
TSPAN5	TSPAN5	tetraspanin 5	Plasma Membrane	other
TUBA1A	TUBA1A	tubulin alpha 1a	Cytoplasm	other
VMP1	VMP1	vacuole membrane protein 1	Plasma Membrane	other
WASF3	WASF3	WAS protein family member 3	Cytoplasm	other
WISP2	WISP2	WNT1 inducible signaling pathway protein 2	Extracellular Space	growth factor

Supplementary Table S5. Genes down-regulated in both oligospermic and azoospermic patients

ID	Symbol	Entrez Gene Name	Location	Type(s)

ACTR1A	ACTR1A	ARP1 actin-related protein 1 homolog A, centractin alpha (yeast)	Cytoplasm	other
BDH1	BDH1	3-hydroxybutyrate dehydrogenase, type 1	Cytoplasm	enzyme
C9orf43	C9orf43	chromosome 9 open reading frame 43	Other	other
CKLF	CKLF	chemokine-like factor	Extracellular Space	cytokine
CMC2	CMC2	C-x(9)-C motif containing 2	Cytoplasm	other
CUL2	CUL2	cullin 2	Nucleus	enzyme
CYC1	CYC1	cytochrome c-1	Cytoplasm	enzyme
EIF6	EIF6	eukaryotic translation initiation factor 6	Cytoplasm	translation regulator
FANK1	FANK1	fibronectin type III and ankyrin repeat domains 1	Nucleus	transcription regulator
GALE	GALE	UDP-galactose-4-epimerase	Cytoplasm	enzyme
GOSR1	GOSR1	golgi SNAP receptor complex member 1	Cytoplasm	transporter
GPN1	GPN1	GPN-loop GTPase 1	Nucleus	transcription regulator
IL4I1	IL4I1	interleukin 4 induced 1	Cytoplasm	enzyme
LRFN4	LRFN4	leucine rich repeat and fibronectin type III domain containing 4	Plasma Membrane	other
MRPL14	MRPL14	mitochondrial ribosomal protein L14	Cytoplasm	other
NACA	NACA	nascent polypeptide-associated complex alpha subunit	Cytoplasm	transcription regulator
PARP4	PARP4	poly(ADP-ribose) polymerase family member 4	Cytoplasm	enzyme
PPP1CC	PPP1CC	protein phosphatase 1, catalytic subunit, gamma isozyme	Nucleus	phosphatase
PSMA4	PSMA4	proteasome subunit alpha 4	Cytoplasm	peptidase
PVRL3	PVRL3	poliovirus receptor-related 3	Plasma Membrane	other
RACGAP1	RACGAP1	Rac GTPase activating protein 1	Cytoplasm	transporter
RAN	RAN	RAN, member RAS oncogene family	Nucleus	enzyme
REEP4	REEP4	receptor accessory protein 4	Cytoplasm	other
SEPT7P2	SEPT7P2	septin 7 pseudogene 2	Other	other
SESN3	SESN3	sestrin 3	Extracellular Space	other
SLC39A3	SLC39A3	solute carrier family 39 (zinc transporter), member 3	Plasma Membrane	transporter
SPA17	SPA17	sperm autoantigenic protein 17	Plasma Membrane	other
SPATA8	SPATA8	spermatogenesis associated 8	Other	other
SPESP1	SPESP1	sperm equatorial segment protein 1	Cytoplasm	other
SUCLG1	SUCLG1	succinate-CoA ligase, alpha subunit	Cytoplasm	enzyme
TMBIM4	TMBIM4	transmembrane BAX inhibitor motif containing 4	Nucleus	other
TMEM219	TMEM219	transmembrane protein 219	Other	other
ZFP91	ZFP91	ZFP91 zinc finger protein	Nucleus	transcription regulator
ZNF541	ZNF541	zinc finger protein 541	Nucleus	other

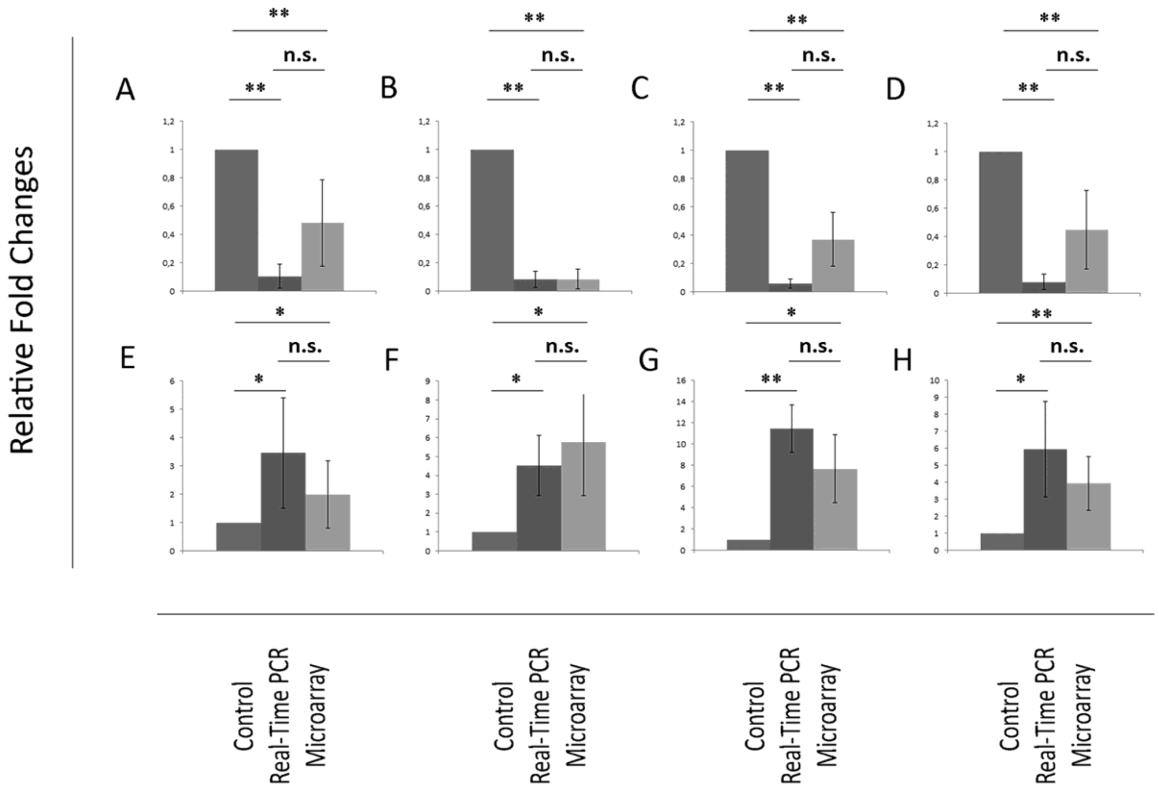

Supplementary Figure S1. Quantitative Real-Time PCR

qRT-PCR on the 8 selected genes CREM (A), AKAP4 (B), LDHC (C), SPA17 (D), BCL2 (E), GSTA (F), JUN (G), and SOD1 (H). qRT-PCR bars show the relative fold changes calculated by ΔΔCt method ± SD. T-test was used to assess the p-Value (* $p<0.05$; ** $p<0.005$). Each gene was analysed in n=3 KS patients and in the control pool RNA (poll from n=3 control subjects). RT-PCR data confirmed microarray data, showing no significant fold changes differences (n.s., Student's T-test) in all analysed transcripts. CREM, LDHC and SPA17 show slight differences between Microarray and qPCR, but they remain statistically not significant.

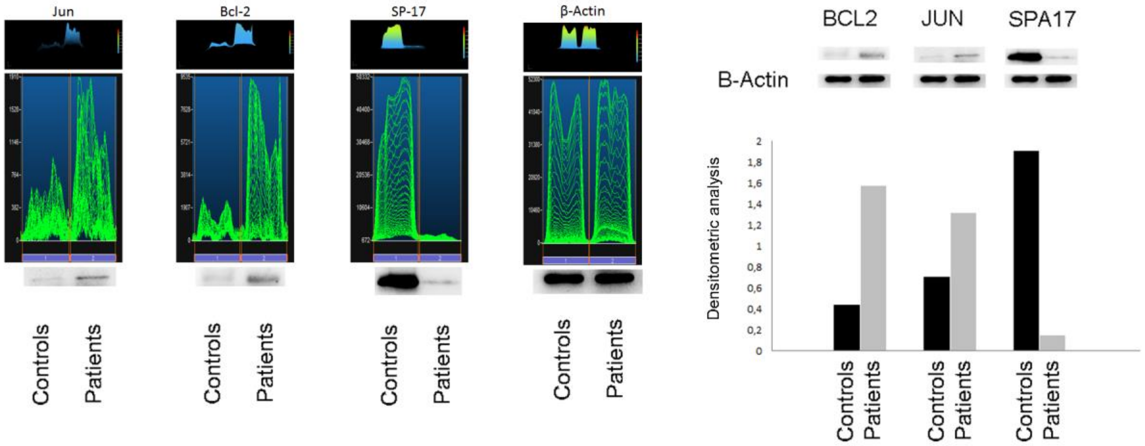

Supplementary Figure S2. Western blot

Western blot analysis of BCL2, JUN and SPA17 proteins from 3 controls and the 3 patients. Proteins for controls and patients were pooled. Protein levels were corrected against β-actin following a densitometric analysis.
